# Aptamer-functionalized EGCG nanocarrier for neutrophil-targeted AKBA delivery promotes anxiety-related fracture healing

**DOI:** 10.1016/j.bioactmat.2026.09.028

**Published:** 2026-09-24

**Authors:** Lizhi Ouyang, Yunhua Zhang, Jiewen Liao, Zhenhe Zhang, Xi He, Ze Lin, Ranyang Tao, Xudong Xie, Fawwaz Al-Smadi, Wu Zhou, Hang Xue, Yiqiang Hu, Dong Yang, Guohui Liu

**Affiliations:** a Department of Orthopedics, Union Hospital, Tongji Medical College, Huazhong University of Science and Technology, 1277 Jiefang Avenue, Wuhan, 430022, People's Republic of China; b Union Hospital, Hospital, Tongji Medical College, Huazhong University of Science and Technology, Wuhan, Hubei, 430030, People's Republic of China; c Department of Ultrasound, Renmin Hospital of Wuhan University, Wuhan, 430060, People's Republic of China; d Department of Bone and Joint Surgery, Department of Orthopedics, Renji Hospital, School of Medicine, Shanghai Jiao Tong University, 145 Shandong Middle Road, Shanghai, 200001, People's Republic of China

**Keywords:** Fracture healing, Anxiety, Neutrophil, Targeted drug delivery system, AKBA

## Abstract

Anxiety is clinically associated with impaired fracture healing, but the underlying immunological mechanism remains unclear, and no targeted therapy is available. In a mouse model of chronic restraint stress (CRS)-induced anxiety with femoral fracture, we observed expansion of TGFβ1^+^CCR5^+^ double-positive neutrophils in the bone marrow. These neutrophils inhibited osteogenic differentiation of bone marrow mesenchymal stem cells (BMSCs) via TGFβ1 secretion, while BMSC-derived CCL5 reciprocally promoted neutrophil activation, forming a detrimental feedback that disrupted fracture repair. Mechanistically, CRS activated the NF-κB–CCR5 axis to upregulate CCR5 and sustain this neutrophil subset. We found that acetyl-11-keto-β-boswellic acid (AKBA) directly bound YAP1, relieved YAP1-mediated suppression of ubiquitin-specific peptidase 31 (USP31), and consequently inhibited NF-κB phosphorylation and CCR5 expression, reducing the pathogenic neutrophil population and restoring BMSCs' osteogenic function. In order to overcome the problems of poor solubility, low bioavailability and tissue non-selectivity of AKBA, we developed a NIMP-R14 aptamer-modified epigallocatechin gallate (EGCG)-coated nanocarrier (AKBA@E-N), which can achieve enhanced uptake and local retention of neutrophils in the acidic microenvironment of the fracture site. In vivo, AKBA@E-N significantly accelerated fracture healing compared with free AKBA. This study reveals a neutrophil-mediated mechanism linking stress to impaired bone repair and provides a targeted nanotherapeutic strategy.

## Highlights

• Anxiety expands TGFβ1^+^CCR5^+^ neutrophils that impair fracture healing.
• AKBA targets YAP1 and suppresses the USP31-NF-κB-CCR5 axis in neutrophils.
• A neutrophil-targeted AKBA@E-N delivery system is developed using NIMP-R14.
• AKBA@E-N restores osteogenesis and accelerates fracture repair in anxious mice.

## Graphical abstract

**Figure:**
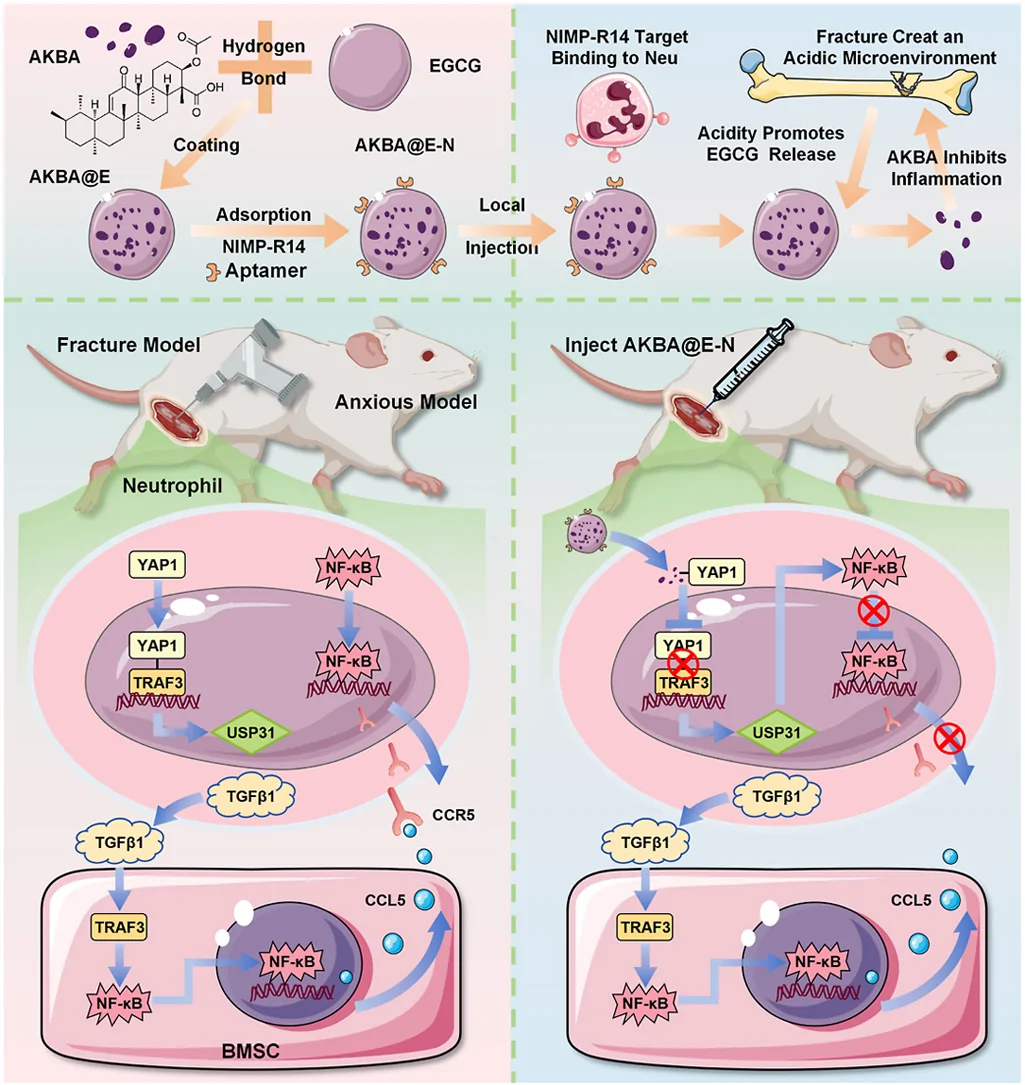

## 1 Introduction

Delayed or non-union bone fracture remains one of the most challenging complications in orthopaedics, and its pathogenesis extends far beyond local tissue damage [[1], [2], [3]]. Epidemiological and clinical evidence consistently indicate that patients with comorbid anxiety or depression exhibit greater bone loss, higher fracture incidence, and protracted healing [[4], [5], [6]]. For these patients, already burdened by psychological distress, impaired fracture repair further increases the risk of permanent disability [[7], [8], [9], [10]], worsens long-term functional outcomes, and escalates healthcare costs—a dual physiological and psychological toll that makes this population a pressing target for clinical intervention [[11], [12], [13], [14], [15], [16], [17], [18]]. Yet the molecular mechanisms by which anxiety modulates fracture healing remain largely undefined, and no targeted therapeutic strategy is currently available.

Fracture healing is initiated by a well-orchestrated inflammatory response. Neutrophils, as the first immune cells to arrive at the injury site and launch the repair program, clear necrotic debris and release inflammatory signals, thereby laying the groundwork for subsequent bone regeneration [[19], [20], [21], [22]]. Their functional orientation largely dictates the ultimate course of repair. Within the functionally heterogeneous neutrophil pool, the TGFβ1^+^CCR5^+^ double-positive subset has recently been identified as a critical regulator of bone metabolism. This subset is the primary source of TGFβ1 in the mouse bone marrow and is aberrantly expanded in age-related bone loss, directly driving reductions in bone mass. Targeted ablation of this population effectively reverses bone loss, underscoring its centrality as a pathological hub in skeletal homeostasis [23]. However, whether anxiety—a highly prevalent negative emotional state in clinical practice—delays fracture healing through aberrant expansion of this bone-specific pathogenic neutrophil subset remains entirely unknown.

To address this question, we employed a mouse model of CRS-induced anxiety combined with femoral fracture and confirmed that anxiety markedly expanded the TGFβ1^+^CCR5^+^ double-positive neutrophil population in the bone marrow, accompanied by overtly impaired fracture healing. This finding positions the pathogenic subset as a potential bridge linking anxiety to defective bone repair. Mechanistically, CRS drives the accumulation of this subset through activation of the NF-κB-CCR5 signaling axis in neutrophils. We next asked whether pharmacological reduction of this subset could rescue healing, and focused on acetyl-11-keto-β-boswellic acid (AKBA) [[24], [25], [26]]. AKBA is a pentacyclic triterpenoid derived from frankincense resin, possessing anti-inflammatory, antioxidant, neuroprotective, and osteogenic-promoting activities, among which its anti-inflammatory and bone-protective properties are particularly relevant to our core question [[27], [28], [29], [30], [31]]. Mechanistic studies revealed that AKBA targets YAP1 to upregulate ubiquitin-specific peptidase 31 (USP31), which in turn suppresses NF-κB phosphorylation and CCR5 expression, thereby significantly reducing the TGFβ1^+^CCR5^+^ neutrophil fraction and restoring the osteogenic differentiation capacity of BMSCs.

However, the poor water solubility, low bioavailability, and lack of tissue selectivity of AKBA severely limit its in vivo efficacy. To overcome these obstacles, we designed a NIMP-R14 aptamer-modified nanocarrier system with an epigallocatechin gallate (EGCG) shell (AKBA@E-N), which enables enhanced neutrophil uptake and microenvironment-responsive release of AKBA. In vivo experiments confirmed that this strategy effectively accelerates fracture healing in anxious mice. These results indicate that targeted elimination of the TGFβ1^+^CCR5^+^ pathogenic neutrophil subset represents a viable route to rescue anxiety-associated fracture delay. Collectively, this study elucidates a novel mechanism underlying anxiety-related delayed fracture healing and offers a strategy with promising translational therapeutic potential (Scheme 1).

**Scheme 1:**
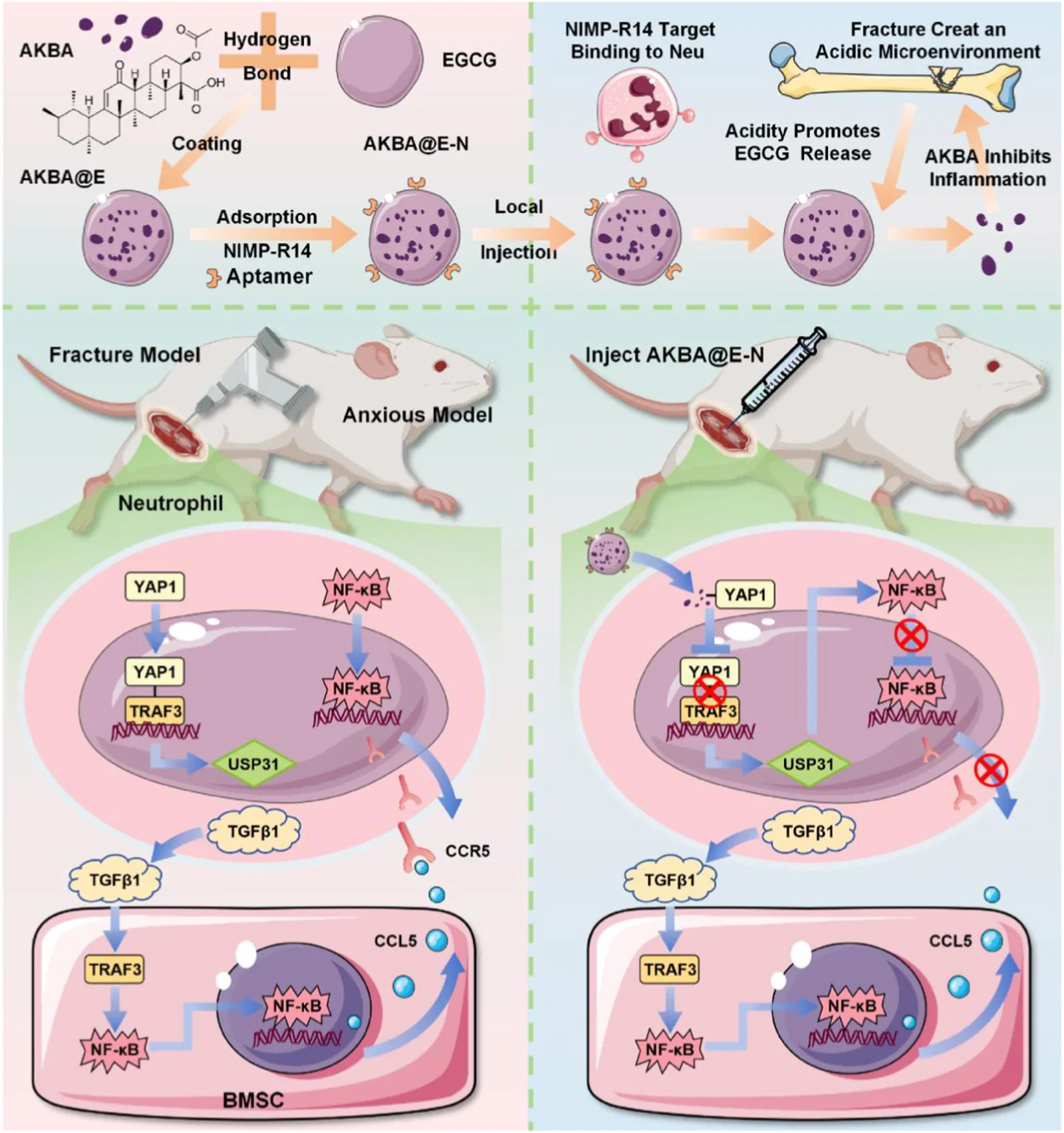
**Mechanism and therapy of anxiety-related delayed fracture healing.** In a mouse model of anxiety combined with fracture, CRS activates the YAP1–USP31–NF-κB–CCR5 signaling axis in neutrophils, leading to abnormal accumulation of TGFβ1^+^CCR5^+^ double-positive neutrophils and excessive TGFβ1 secretion. TGFβ1 suppresses TRAF3 expression and activates the NF-κB–CCL5 pathway in BMSCs, forming a positive feedback loop that disturbs bone homeostasis and delays fracture healing. The AKBA@E-N delivery system constructed in this study uses EGCG as the carrier, loads AKBA, and modifies it with the NIMP-R14 aptamer, which can be released in the acidic microenvironment of the fracture site to achieve local retention and enhance neutrophil uptake. By inhibiting the above-mentioned signaling axis, AKBA can reverse healing impairment and promote bone repair.

## 2 Materials and methods

### 2.1 Mouse model

Seven-week-old specific pathogen-free (SPF) male C57BL/6J mice with matched body weight were used in this study and housed under standard laboratory conditions. A CRS protocol was applied to establish the mouse anxiety model, which is widely used for studying anxiety and depression related psychosomatic disorders. Before modeling, all mice underwent an open-field test to assess spontaneous locomotor activity, and animals with activity levels outside the 95% confidence interval were excluded. The screened mice were randomly assigned to the control and CRS groups. Mice in the control group received routine feeding, while those in the CRS group underwent 4 h of daily restraint at 12:00 for 14 consecutive days. For restraint, mice were placed head-down into 50 mL centrifuge tubes perforated with dozens of 2 mm ventilation holes, and the tubes were fixed horizontally on a flat surface to ensure ventilation and stable restraint. Body weight measurement and behavioral tests were performed on day 14 to verify the successful establishment of the anxiety model.

After the CRS model was successfully constructed, all mice received unilateral femoral fracture surgery. Mice were anesthetized by intraperitoneal injection of 1% sodium pentobarbital. After skin preparation and disinfection of the right hind limb, a longitudinal incision was made at the medial mid-femur, and soft tissues were bluntly dissected to expose the femur. A stable closed transverse fracture was created at the femoral mid-shaft with bone rongeurs while preserving the periosteum, followed by intramedullary fixation using a 0.3–0.5 mm Kirschner wire. The incision was closed layer by layer after confirming adequate alignment of the fracture ends. Postoperatively, mice were housed individually with routine wound care and anti-infective treatment. An X-ray was performed to confirm a clear fracture line, stable internal fixation, and no obvious displacement, indicating successful fracture modeling. Mice were monitored daily for wound healing, food/water intake, limb mobility, and signs of infection. Animals with wound suppuration, severe fracture displacement, or inability to feed were immediately euthanized per predefined humane endpoints.

Mice meeting any of the following criteria were immediately humanely euthanized: (1) body weight loss >20% within 7 days; (2) severe wound infection with tissue necrosis; (3) complete loss of ambulatory function due to fracture malposition; (4) persistent pain unresponsive to analgesic treatment; (5) moribund state with hypothermia, unresponsiveness, or respiratory depression. At the experimental endpoint or when humane endpoints were triggered, mice were first deeply anesthetized with 5% isoflurane inhalation, followed by intraperitoneal injection of an overdose of sodium pentobarbital (150 mg/kg). Death was confirmed by permanent cessation of heartbeat and respiration, and tissue samples were collected immediately thereafter.

Following CRS exposure, peripheral blood specimens were collected on days 0, 7, 14, and 21. Sampling was conducted during the midpoint of the mice's nocturnal inactive phase. Within this time window, basal corticosterone (CORT) and norepinephrine (NE) concentrations remain relatively steady, facilitating sensitive identification of stress-provoked hormonal alterations (n = 6 per group; 0.8 ± 0.1 mL of blood collected per mouse). Fresh whole blood was immediately centrifuged at 3000 g for 10 min under 4 °C to isolate serum for subsequent hormone measurement. Serum adrenocorticotropic hormone (ACTH), corticosterone, epinephrine (EPI), and NE concentrations were quantified via commercially available ELISA kits. Assay kits were obtained from Aifang Biotech (Changsha, China), including CORT (AF2061-A) and NE (AF2533-A). All ELISA procedures were performed in strict accordance with the manufacturer's protocols.

### 2.2 Micro-computed tomography (micro-CT) analysis

At 21 days after fracture surgery, all mice were euthanized via intraperitoneal injection of 150 mg/kg sodium pentobarbital. The fractured right femur was harvested, stripped of adherent soft tissues, fixed in 4% paraformaldehyde for 24 h, and stored in phosphate-buffered saline at 4 °C prior to micro-computed tomography (micro-CT) scanning.

(1) Micro-CT scanning parameters. Scanning was performed on a desktop micro-CT system with consistent parameter settings across all samples: tube voltage of 80 kV, tube current of 100 μA, isotropic voxel resolution of 10 μm, 0.5 mm aluminum filter for beam hardening reduction, 360° full rotation with a 0.5° rotation step, and 300 ms exposure time per frame.
(2) Image reconstruction and calibration. Raw projection data were reconstructed into serial transverse 2D slices using the manufacturer's dedicated reconstruction software, with ring artifact correction and beam hardening correction enabled during reconstruction. A standard hydroxyapatite (HA) phantom with two predefined mineral density gradients was scanned under identical parameters to calibrate grayscale values to absolute bone mineral density (BMD), ensuring quantitative comparability across all samples.
(3) Definition of region of interest (ROI) and anatomical boundaries. The ROI for quantitative callus analysis was strictly delineated using consistent anatomical landmarks. First, the midpoint of the fracture gap was identified on consecutive coronal and sagittal reformatted slices as the central anatomical reference. The ROI was then defined as a 3 mm-long cylindrical volume extending 1.5 mm proximally and 1.5 mm distally from the fracture line, which fully encompassed all periosteal and endosteal callus tissue. The intact mature cortical bone at both ends far from the fracture site was deliberately excluded from the ROI to avoid interference from native bone.
(4) Thresholding strategy and quantitative analysis. A fixed global thresholding approach was adopted to segment mineralized bone tissue from surrounding soft tissue, marrow space, and non-mineralized callus. Based on HA phantom calibration, the grayscale threshold corresponding to 700 Hounsfield Units (HU) was uniformly applied to all samples for mineralized callus segmentation. All ROI delineation and threshold setting were performed by two independent researchers in a blinded manner to minimize measurement bias.

Three-dimensional reconstruction of the ROI was generated for morphological visualization, and three core parameters were quantified to evaluate fracture healing: (1) bone volume fraction (BV/TV, %), calculated as the ratio of mineralized callus volume to total tissue volume within the ROI; (2) bone mineral density (BMD, g/cm^3^), calibrated to reflect the mineralization level of newly formed bone. All quantitative data were exported from the built-in analysis module of the micro-CT system.

### 2.3 Behavioral tests

All behavioral tests were performed on the same cohort of mice after 14 days of CRS exposure and before fracture surgery, following a standardized protocol to minimize cross-paradigm stress carryover effects. Tests were administered in ascending order of stress intensity, with a minimum 24-h rest interval between independent assays. All tests were conducted during the late light phase under consistent illumination (200 lux) and temperature (22 ± 1 °C). The apparatus was thoroughly cleaned with 75% ethanol between animals to eliminate residual olfactory cues, and all behavioral data were quantified by two blinded researchers. The tests were performed in the following order: (1) Open Field Test (OFT) on Day 14, (2) Elevated Plus Maze (EPM) on Day 15, (3) Sucrose Preference Test (SPT) on Days 16–18 (with 48-h habituation and 12-h food deprivation prior to testing), (4) Forced Swimming Test (FST) on Day 19 (with a 15-min pretest on Day 18), and (5) Tail Suspension Test (TST) on Day 20. This sequential order ensures that anxiety-related tests (OFT, EPM) are completed before the more stressful depression-related tests (FST, TST), thereby minimizing carryover stress effects. Using one week as a cycle, OFT was repeated in sequence on days 21 and 28, and all behavioral tests were performed across three cycles.

**Elevated Plus Maze (EPM):** Anxiety-like behaviors were assessed using an elevated plus maze with 30–35 cm-long arms, 5 cm wide, and 50–60 cm high, under 100–300 lux illumination. Mice were acclimated for 30 min before testing and then placed in the maze center, facing an open arm. Activity was recorded for 5 min, and anxiety levels were quantified as the percentage of time spent and entries in the open arms. The maze was sanitized with 75% ethanol between tests, and all assays were performed during the light phase.

**Open Field Test (OFT):** Locomotor activity and anxiety were evaluated in a 500 mm × 500 mm × 415 mm enclosed arena, with behavior recorded by an overhead camera. Mice were acclimated for 7 days with daily handling, then transferred to the test room 1 h before experimentation. After placement in the arena center, movement was tracked for 15 min. Total distance traveled, central zone entries, and residence time were analyzed automatically in a double-blinded manner. Abnormally active mice were excluded, and OFT was conducted first.

**Forced Swimming Test (FST):** Depressive-like behaviors were measured over two days. On day 1, mice swam for 15 min in water (25–30 °C, 10 cm depth) and were dried before being returned to their cages. On day 2, mice were tested for 6 min, and immobility time during the last 4 min was recorded as a marker of depressive behavior. Tests were performed at fixed times in the same season to control for environmental variables.

**Tail Suspension Test (TST):** Mice were acclimated for 30 min, then suspended 50 cm above the floor by adhesive tape 1 cm from the tail tip. Behavior was recorded for 6 min under dim light, and immobility time in the last 4 min was quantified to indicate depressive-like behaviors. Assays were performed at a fixed circadian time to avoid timing bias.

**Sucrose Preference Test (SPT):** Individually housed mice were acclimated to 1% sucrose solution and distilled water for 48 h, with bottle positions swapped every 12 h. After 12 h of food deprivation, liquid consumption was measured at 0, 12, 24, and 36 h. The sucrose preference coefficient was calculated as (sucrose intake/total fluid intake) × 100%, with lower values indicating anhedonia.

### 2.4 Isolation of neutrophils

Neutrophils were isolated from mouse bone marrow using a commercial mouse bone marrow neutrophil isolation kit (Haoyang Bio). All procedures were performed in a sterile environment at 18–22 °C and completed within 2 h after sample collection. Following euthanasia, mouse femurs or tibias were dissected, and both ends of the bones were transected. Bone marrow was flushed from the medullary cavity using homogenate washing buffer to obtain a cell suspension. The suspension was filtered through a 70 μm cell strainer and centrifuged at 450×g for 10 min. The resulting cell pellet was resuspended in red blood cell lysis buffer to a final concentration of 2×10^8^–1×10^9^ cells/mL. The 80% isolation solution was prepared by mixing Isolation Solution 1 and tissue sample dilution buffer at a ratio of 4:1. According to the sample volume (0.5–3.0 mL), Isolation Solution 1 and the prepared 80% isolation solution were sequentially added into sterile centrifuge tubes to form a density gradient. The cell suspension was slowly overlaid onto the gradient surface, then centrifuged at 450–600×g for 30 min. The third milky-white, ring-shaped neutrophil layer was carefully aspirated. The harvested neutrophils were washed with 5–10 mL of washing buffer and subjected to two sequential centrifugation cycles (400×g for 10 min, then 250×g for 10 min). Finally, the cells were resuspended in 0.5 mL of washing buffer or the desired experimental solution.

### 2.5 Magnetic-activated cell sorting (MACS) of neutrophils

TGFβ1^+^CCR5^+^ double-positive neutrophils were isolated from mouse bone marrow by a two-step MACS procedure following a previously established protocol [23]. Briefly, bone marrow cells were sequentially incubated with biotinylated anti-CCR5 and anti-TGFβ1 antibodies (BioLegend), followed by anti-biotin microbeads (Miltenyi Biotec) and positive selection using LS columns (Miltenyi Biotec). All procedures were performed at 4 °C to preserve cell viability.

### 2.6 Co-culture of neutrophils and BMSCs

An in vitro co-culture system of primary neutrophils and BMSCs were established using Transwell chambers. Primary neutrophils were seeded in the upper chamber, and BMSCs were cultured in the lower chamber. Before co-culture, BMSCs were plated at a density of 5 × 10^4^ cells per well and cultured in complete medium until adherence and confluence. Primary mouse neutrophils were isolated by MACS, and the cell concentration was adjusted immediately after sorting for seeding into the upper Transwell chambers. The chambers were then transferred to the culture plates pre-seeded with BMSCs, and an equal volume of medium was added to maintain consistent liquid levels, initiating co-culture. Given that primary mouse neutrophils lack proliferative capacity in vitro and undergo rapid apoptosis within 24 h, the total co-culture duration was strictly controlled at 21.5 h. To ensure experimental stability, primary neutrophils were freshly isolated daily, and those in the upper Transwell chambers were promptly replaced after MACS to avoid interference from cell apoptosis.

### 2.7 Immunofluorescence staining and confocal microscopy

Purified primary neutrophils obtained by magnetic-activated cell sorting were collected in EP tubes and fixed with 4% paraformaldehyde at room temperature for 15 min. After washing, cells were permeabilized with 0.1% Triton X-100 at room temperature and then blocked in PBS containing 5% bovine serum albumin (BSA) for 1 h to prevent non-specific binding. Cells were incubated with diluted rabbit anti-mouse CCR5 primary antibody (Servicebio, GB11504-100, 1:200 in PBS containing 1% BSA) at 4 °C overnight. The next day, a fluorescently labeled secondary antibody (Cy3-labeled goat anti-rabbit IgG, 1:200) was added and incubated for 50 min at room temperature in the dark. After three washes with PBS for 5 min each, rabbit anti-mouse TGFβ1 primary antibody (Proteintech, Cat No. 81746-2-RR, 1:150) was labeled with FITC using the FITC labeling kit (Proteintech, Cat No.: KFA501) and then incubated with cells for 50 min. Subsequently, cells were stained with DAPI staining solution for 15 min at room temperature in the dark to label nuclei. An anti-fade mounting medium was added, and 10 μL of the cell suspension was placed on a glass slide and covered with a coverslip. Images were captured with a laser-scanning confocal microscope using a 100× oil-immersion objective, with excitation wavelengths of 488 nm (FITC), 555 nm (Cy3), and 405 nm (DAPI). Z-stack scanning was performed to confirm fluorescence localization. The co-expression of TGFβ1 and CCR5 was analyzed using merged images, and the proportion of double-positive cells was quantified in ImageJ. All procedures were performed in the dark. A negative control without primary antibody was set to exclude non-specific fluorescence interference.

### 2.8 Quantitative real-time PCR (qrt-PCR)

Five distinct sample types were prepared for qRT-PCR quantification: MACS-sorted bone marrow neutrophils, in vitro-cultured BMSCs, in vivo fracture callus tissues, Hoxb8 cells, and HL-60 cells with specific sampling sites and isolation procedures described separately below.

(1) MACS-sorted TGFβ1^+^CCR5^+^ neutrophils (for detection of Usp31, Ccr5, Tgfβ1 and inflammatory axis genes). After euthanasia of mice, bilateral femurs and tibiae were sterilely dissected as the exact sampling sites of bone marrow. Both bone epiphyseal ends were cut off, and bone marrow was flushed out with pre-cooled PBS to prepare a single-cell suspension. TGFβ1^+^CCR5^+^ double-positive neutrophils were purified via two-step MACS separation as described in Section 4. Purified cell pellets were collected by centrifugation and immediately lysed with TRIzol reagent for total RNA extraction. Only cell populations with sorting purity ≥78% were subjected to subsequent transcriptional detection.
(2) In vitro co-cultured BMSCs (for detection of osteogenic markers *Runx2, Col1a1, Bglap*). BMSCs grown on the lower chamber of Transwell co-culture systems were used without in vivo tissue sampling. Culture medium was discarded, and adherent BMSCs were washed three times with pre-cooled PBS, scraped off, and lysed with TRIzol to isolate cellular RNA.
(3) Fracture callus tissues from in vivo mouse models (for in vivo osteogenic gene quantification). The sampling site was limited to newly formed callus tissue within 1 mm above and below the femoral fracture line, with mature cortical bone at both fracture ends completely excluded. Fresh callus was dissected under a stereomicroscope immediately after animal sacrifice, snap-frozen in liquid nitrogen, and ground into powder prior to TRIzol-mediated total RNA extraction.
(4) Hoxb8 cells. After washing with PBS, it was used for subsequent operations.
(5) HL-60 cells. After washing with PBS, it was used for subsequent operations.

After RNA extraction, the purity and integrity of total RNA were verified by Nanodrop spectrophotometry (A260/A280 = 1.8–2.1) and agarose gel electrophoresis to exclude RNA degradation. Qualified RNA samples were reverse-transcribed into complementary DNA (cDNA) using an Invitrogen reverse transcription kit following manufacturer's instructions. Quantitative real-time PCR was performed on a Vazymebiotech qPCR instrument with gene-specific primers listed in the table, and β-actin was set as the housekeeping reference gene for all five sample categories. Each biological sample was arranged with 3 technical replicates, and all experiments were independently repeated for 3 biological batches.

The primer sequences were listed as follows Table 1.

**Table 1:** Primer sequences.

Genes		Primer sequences
*β-actin*	Forward	5′-TGAGCTGCGTTTTACACCCT-3′
	Reverse	5′-TTTGGGGGATGTTTGCTCCA-3′
*Runx2*	Forward	5′-ATGCTTCATTCGCCTCACAAA-3′
	Reverse	5′-GCACTCACTGACTCGGTTGG-3′
*Col1a1*	Forward	5′-GCTCCTCTTAGGGGCCACT-3′
	Reverse	5′-CCACGTCTCACCATTGGGG-3′
*Bglap*	Forward	5′-CTGACCTCACAGATCCCAAGC-3′
	Reverse	5′-TGGTCTGATAGCTCGTCACAAG-3′
*Usp31*	Forward	5′-CCTGTATGCTGTGTGCAATCACC-3′
	Reverse	5′-TCGCTGTCATCGAAGCAGTACC-3′
*Traf3*	Forward	5′-AGAGAAGGGCTTGATGGTGC-3′
	Reverse	5′-ACATGATGGCATGAAGCCCA-3′
*Ccl5*	Forward	5′-TGCTCCAATCTTGCAGTCGT-3′
	Reverse	5′-GCAAGCAATGACAGGGAAGC-3′

### 2.9 Western Blotting (WB)

Three independent sample sources were used for western blot detection: MACS-purified bone marrow neutrophils, in vitro co-cultured BMSCs, and fresh fracture callus tissues harvested from mouse femurs, with defined sampling regions and lysis protocols for each.

(1) MACS-sorted TGFβ1^+^CCR5^+^ bone marrow neutrophils (target proteins: YAP1, USP31, p-p65, total p65, CCR5, TGFβ1). Bone marrow was flushed from the bilateral femur and tibia marrow cavities, and TGFβ1^+^CCR5^+^ neutrophils were enriched by magnetic-activated cell sorting as described above. Cell precipitates were resuspended in pre-cooled RIPA lysis buffer supplemented with complete protease inhibitor cocktail and phosphatase inhibitor cocktail, followed by 30 min ice incubation to extract total cellular protein.
(2) Transwell co-cultured BMSCs (osteogenic proteins: COL1A1, OCN, RUNX2). Adherent BMSCs on the lower layer of co-culture inserts were washed with cold PBS, scraped, and fully lysed with inhibitor-containing RIPA buffer on ice for protein acquisition.
(3) Femoral fracture callus tissues (in vivo osteogenic protein quantification). Only nascent callus tissue within 1 mm upstream and downstream of the fracture site was collected; normal cortical bone far from the fracture line was discarded. Frozen callus powder was homogenized in RIPA lysis buffer with protease/phosphatase inhibitors to extract total tissue protein.

The protein concentration of each lysate was normalized via the BCA assay to guarantee equal loading. Equal amounts of total protein were separated by gradient sodium dodecyl sulfate–polyacrylamide gel electrophoresis (SDS-PAGE, 8–12% gel concentration adjusted according to target molecular weight), then wet-transferred onto polyvinylidene difluoride (PVDF) membranes under constant 300 mA current. Membranes were blocked at room temperature for 1 h: 5% skimmed milk was used for total protein detection, while 5% bovine serum albumin (BSA) was applied for phosphorylated protein (p-p65) detection to reduce non-specific binding. The corresponding primary antibodies were incubated with the membranes at 4 °C overnight. After thorough washing with TBST buffer, HRP labeled secondary antibodies were added and incubated at room temperature for 1 h. Target protein bands were visualized via a chemiluminescence imaging system. GAPDH or α-Tubulin was used as the internal reference protein for both cellular and tissue samples. The relative gray value of each target band was quantified using ImageJ software and normalized to the corresponding GAPDH or α-Tubulin band intensity. All western blot assays were performed in three independent biological replicates.

### 2.10 Alizarin Red staining

BMSCs after 14 days of co-culture were rinsed twice gently with PBS after discarding the medium. Cells were fixed with 4% paraformaldehyde at room temperature for 15 min and washed three times with PBS. Then, the cells were stained with Alizarin Red staining solution (pH 8.3) at room temperature for 30 min, followed by three washes with distilled water to remove excess dye. Mineralized nodules were observed and photographed under an inverted microscope. The percentage of mineralized nodule area was quantified using ImageJ, and all experiments were repeated in triplicate.

### 2.11 Alkaline phosphatase (ALP) staining

BMSCs after 7 days of co-culture were washed twice with PBS after removing the medium, fixed with 4% paraformaldehyde at room temperature for 15 min, and rinsed three times with PBS. ALP staining working solution was added, and the cells were incubated at 37 °C for 1 h in the dark. Three washes with distilled water terminated staining. Stained cells were observed and photographed under an inverted microscope. The percentage of positively stained area was quantified with ImageJ software to evaluate ALP activity, and all assays were performed in triplicate.

### 2.12 Hematoxylin and eosin (HE) staining and Masson's trichrome staining

Callus tissues at the mouse fracture site were harvested, fixed in 4% paraformaldehyde, dehydrated through a graded ethanol series, and embedded in paraffin for serial sectioning. The sections were sent to a professional testing company for HE staining and Masson's trichrome staining. After staining, the sections were examined under a light microscope, with focus on callus morphology, distribution of newly formed bone tissue, and cell infiltration. Photographs were taken for histological analysis. The percentage of mature bone (red-stained area) was evaluated to assess bone maturity and collagen deposition at the fracture site.

### 2.13 Material synthesis

First, 0.51 mg of AKBA powder was dissolved in methanol to prepare an AKBA/methanol solution at a final concentration of 1 mmol/L. The solution (100 μL) was then slowly added dropwise to 900 μL of water under vigorous stirring at 1000 rpm at room temperature using a centrifuge (Eppendorf, 5424R, Hamburg, Germany). The mixture was subjected to ultrasonication (500 W, 40 kHz) for 20 min, and the methanol was removed under a nitrogen stream. The solution was centrifuged at 3000 rpm for 10 min (Eppendorf), and the precipitate was discarded to obtain the AKBA nanoparticle solution. EGCG was purchased from Aladdin Reagent Co., Ltd. (Beijing, China). NIMP-R14-Apt was obtained from Sangon Biotech Co., Ltd. (Shanghai, China). EGCG solutions of varying concentrations (20%, 15%, 10%, and 5%) were prepared in water and added to the 10 μM AKBA nanoparticle solution. The mixture was stirred at 1000 rpm for 10 min to generate EGCG-coated AKBA nanoparticles (AKBA@E). Subsequently, 10 μL of NIMP-R14-Apt (10 mmol/L) was added dropwise to 990 μL of the AKBA@E solution, and the mixture was stirred at 500 rpm overnight to obtain AKBA@E-N.

AKBA@E-N was characterized in this study by TEM morphology, DLS particle size and Zeta potential, XPS/Raman/FTIR spectroscopy, drug loading, encapsulation efficiency, and pH-dependent release. The following parameters were not evaluated experimentally: aptamer surface density, EGCG coating thickness, serum stability, long-term storage stability, and formal lot-to-lot reproducibility. The NIMP-R14 aptamer was linked by physical adsorption, and its presence was qualitatively confirmed by spectroscopic characterization; aptamer density was not quantified. All formulations were prepared fresh prior to each experiment, and no storage stability claims were made.

### 2.14 Flow cytometry analysis

Bone marrow single-cell suspensions were prepared as described above, and cell density was adjusted to 1 × 10^6^ cells per tube. For surface marker staining, cells were pre-incubated with Fc receptor block for 10 min at 4 °C to minimize non-specific antibody binding, then stained with fluorophore-conjugated anti-CD11b and anti-Ly6G antibodies for 30 min at 4 °C in the dark. For intracellular TGFβ1 detection, cells were fixed and permeabilized, followed by incubation with anti-TGFβ1 and anti-CCR5 antibodies for 40 min at 4 °C.

Single-stained compensation beads were prepared for each fluorochrome to generate a unified compensation matrix, which was uniformly applied to all samples to correct for fluorescence spillover between detection channels. Both isotype controls and fluorescence minus one (FMO) controls were set up to ensure accurate and unbiased gating. Isotype-matched antibodies were processed under identical fixation and permeabilization conditions to define the baseline of non-specific antibody binding. FMO controls, in which one target fluorophore was omitted from the full staining panel, were used to demarcate the positive gate boundaries for CCR5 and TGFβ1 double-positive populations. A standardized sequential gating strategy was consistently applied to all samples: Total leukocytes were first gated on the FSC-A/SSC-A scatter plot to exclude cellular debris and particulate impurities; Single cells were selected via the FSC-H/FSC-A plot to eliminate cell doublets and aggregates; CD11b^+^Ly6G^+^ mature neutrophils were further identified within the single-cell population; The TGFβ1^+^CCR5^+^ pathogenic neutrophil subset was finally gated and quantified within the mature neutrophil gate. All samples were acquired on a BD LSRFortessa flow cytometer and analyzed with FlowJo v10 software.

### 2.15 Material characterisation

The morphology of the nanoparticles was characterized by scanning electron microscopy (SEM; FEI, Verios G4 UC, Hillsborough, OR, USA) to assess their physical properties. The average particle size and zeta potential of the obtained products were determined by dynamic light scattering (DLS; Malvern Panalytical, Nano-S90, Malvern, UK) to evaluate the size distribution. Sample preparation was performed following a previously reported method.

### 2.16 Biocompatibility of materials

**Hemolysis Test:** To verify the biocompatibility of AKBA@E-N, mouse peripheral blood was collected and placed in a conical flask containing glass beads, followed by shaking for 10 min. Alternatively, the blood was stirred with a glass rod to remove fibrinogen and prepare defibrinated blood. Red blood cells were washed 2–3 times with 0.9% sodium chloride solution to prepare a 4% cell suspension. Triton X-100, PBS, AKBA, EGCG, and AKBA@E-N were added separately, and the mixtures were incubated in an incubator at 37 °C with 60 rpm for 1 h. Hemolytic phenomena were observed and recorded. n = 3.

**In Vivo Histological Evaluation:** (1) Control, (2) AKBA, (3) EGCG, (4) AKBA@E-N. were applied to the fracture site of 7-week-old male C57BL/6J mice, respectively. After 12 days, major organs, including the heart, liver, spleen, lung, and kidney, were harvested and fixed. Tissue sections were stained with hematoxylin and eosin (HE) and scanned using an EPSON V600 digital scanner. n = 3.

### 2.17 In vivo therapeutic evaluation

For in vivo pharmacodynamic evaluation, mice were randomly assigned to seven groups (n = 6 per group): Control, CRS, CRS + AKBA, CRS + EGCG, CRS+@E-N, CRS + AKBA@E, and CRS + AKBA@E-N. Immediately after fracture surgery and intramedullary fixation, 20 μL of the respective formulation was locally injected into the fracture site (periosteal and surrounding soft tissue space) before wound closure. The AKBA dose was standardized at 5 mg/kg body weight for both the free AKBA and AKBA@E-N groups, based on our in vitro CCK-8 results and previous reports demonstrating the effective dose range of AKBA in rodent models [32,33]. Free AKBA was dissolved in saline containing 2% DMSO, while AKBA@E-N was administered as a nanoparticle suspension at the equivalent AKBA concentration. Single administration was performed at day 0 post-fracture, leveraging the sustained-release property of the EGCG coating to maintain local drug levels over the subsequent healing period.

### 2.18 In vivo microenvironment pH

Mice in the control and CRS groups were anesthetized, and the femoral fracture site, along with its internal microenvironment, was fully surgically exposed to enable repeated pH assessment of the fracture microenvironment following a standardized protocol. Local microenvironmental pH was quantified using a microelectrode pH meter. The sensing tip of the microelectrode was positioned at three randomly selected sites on the fracture surface, and the mean value of stabilized readings was adopted for subsequent data analysis. Protein residues on the sensor surface were removed by immersing the electrode in an enzymatic detergent for approximately 2 min after every single measurement. An extended 30-min decontamination procedure was performed upon completion of each full testing session, followed by thorough rinsing with deionized water. The electrode was replaced promptly when a decline in its response speed was observed.

### 2.19 Determination of drug loading capacity and encapsulation efficiency

The indirect method was employed, whereby a specific quantity of the AKBA@E-N nanoparticle formulation (containing a known total drug amount) was placed in an ultrafiltration centrifuge tube. Centrifugation was carried out to separate the free drug from the nanoparticles, and the filtrate (containing unencapsulated free AKBA) was collected. High-performance liquid chromatography (HPLC) was used to determine the concentration of free drug in the filtrate at the characteristic absorption wavelength of AKBA. Simultaneously, an equal volume of the unseparated formulation was taken; the nanostructure was disrupted using an organic solvent (e.g., methanol) before the total drug concentration was determined. The drug loading capacity and encapsulation efficiency were calculated using the formulae “Drug loading capacity (%) = (Total drug content − Free drug content)/Total mass of nanoparticles × 100%” and “Encapsulation efficiency (%) = (Total drug content − Free drug content)/total drug amount × 100%’ to calculate drug loading capacity and encapsulation efficiency, respectively. Three replicate samples were prepared for each group, and the results are expressed as the mean ± SD [34].

### 2.20 Pharmacokinetics

A CRS-induced anxiety-related fracture model was established. The groups—the AKBA group, the AKBA@E group, and the AKBA@E-N group—received local injections at the fracture site, with all groups receiving the same dose. Drug concentrations at the fracture site and in serum were measured at 0.5 h, 1 h, 2 h, 4 h, 8 h, 12 h, 24 h, 48 h, and 72 h. Curves were plotted using GraphPad Prism, and Cmax (peak concentration, ng/mL), Tmax (time to peak, h), and AUC_0-t_ (area under the concentration-time curve, from 0 to the final blood sampling point).

### 2.21 Immunofluorescence verification of targeted delivery function in vivo

AKBA@E-N, AKBA@E, and free AKBA were each fluorescently labeled with Cy3 and administered topically to the fracture site. After 24 h, callus tissue was harvested from the fracture site, and frozen sections were prepared. Using a FITC-labeled anti-LY6G antibody, the degree of colocalization between the Cy3 fluorescence signal and the FITC (LY6G) signal was observed and quantified using a confocal laser scanning microscope.

### 2.22 Flow cytometry to verify targeted delivery function

Bone marrow cells were isolated from mice in each group and analyzed by flow cytometry. The AKBA was labeled with FITC, CD11b with PE, and LY6G with PerCP/Cy5.5. After identifying the neutrophil population (CD11b^+^LY6G^+^), the mean fluorescence intensity (MFI) of intracellular FITC was measured [35].

### 2.23 Materials physics characterisation and testing

XPS X-ray photoelectron spectroscopy: Freeze-dried EGCG, AKBA@E, and AKBA@E-N nanoparticle samples were ground into a fine powder, spread evenly onto conductive carbon tape, and prepared as test specimens. Surface elemental analysis was performed using an X-ray photoelectron spectrometer, with monochromatic Al Kα radiation (hν = 1486.6 eV) as the excitation source, an operating voltage of 15 kV, and a beam current of 15 mA. Full spectrum scan range is 0-1350eV, high-resolution spectra were acquired for the C1s and O1s core levels to analyze the chemical states and bonding environments of carbon and oxygen atoms [36,37].

Raman: Place the freeze-dried EGCG, AKBA@E, and AKBA@E-N nanoparticle samples onto a microscope slide or quartz slide, and gently press them flat to ensure the sample surface is smooth and uniform. Perform the analysis using a confocal Raman spectrometer, employing a laser with a wavelength of 532 nm or 785 nm as the excitation source (depending on the sample's fluorescence background), with the laser power set to 1–10 mW to avoid thermal damage to the sample. Set the spectral scanning range to 400–2000 cm^−1^ (the fingerprint region), as this region contains a wealth of molecular vibrational information and effectively reflects the chemical composition and structural characteristics of the sample. Select 3-5 random points from each sample for data acquisition to ensure the representativeness of the spectra [38].

FTIR: Freeze-dried samples of EGCG, AKBA, AKBA@E, and AKBA@E-N nanoparticles were each mixed with dried KBr powder in a mass ratio of approximately 1:100 and thoroughly ground in an agate mortar to a fine, uniform powder, ensuring even sample dispersion. Using a tablet press, the mixed powder was compressed at a pressure of approximately 5–10 MPa to form transparent discs with a diameter of approximately 13 mm and a thickness of approximately 1 mm. Spectra were acquired in transmission mode using a Fourier transform infrared spectrometer [39].

These spectroscopic analyses were used to confirm the presence of the EGCG coating and the adsorption aptamer. Coating thickness and aptamer surface density were not quantified in this study.

### 2.24 Bioinformatics analysis

Total RNA was extracted from target cell samples and subjected to quality control. Qualified samples were sent to Novogene Co., Ltd. for transcriptome sequencing on the Illumina NovaSeq 6000 platform. Raw data were processed with quality control and aligned to the mouse mm10 genome. Differentially expressed genes (DEGs) were screened using DESeq2 software with thresholds of |log2FC| > 1 and padj < 0.05. Volcano plots were generated using the Bioinformatics online tool to visualize DEG expression patterns. KEGG pathway enrichment analysis for the mouse species was performed (padj < 0.05), and enrichment bubble plots were produced. Core DEGs were uploaded to the STRING database (mouse, confidence score = 0.7) to construct a protein–protein interaction network, which was visualized as a chord diagram. Molecular docking was performed using Autodock Vina. The structures of AKBA and core receptor proteins were processed and docked with PyMOL, ChemDraw, and AutoDock Tools. The binding energy was calculated, the optimal conformation was selected, and the binding sites and interaction patterns were visualized.

### 2.25 Antibacterial evaluation

*Escherichia coli* (*Ecoli*) and methicillin-resistant *Staphylococcus aureus* (MRSA) were used to test the bacteriostatic potential; PBS was added to the upper and lower chambers of the Transwell. Then, PBS, AKBA, NIMP-R14, EGCG, @E-N, and AKBA@E-N were added to the upper chamber of the Transwell, respectively, and the pH value was uniformly adjusted to 6.79. After 4 h of release, the lower-chamber solution was incubated with bacteria for 12 h to test its antibacterial activity. 1) The bacteria were inoculated by spreading them evenly on the surface of the agar medium. 2) The lower chamber solution was used to spread the bacteria on the medium flatly and incubated at 37 °C for 2 h. The solution was diluted 500-fold and spread evenly on LB agar plates. 3) After incubation at 37 °C for 24 h, pictures were taken with an EPSON V600 digital scanner.

For scanning electron microscopy (SEM) analysis, 100 μL of bacterial suspension (1 × 10^8^ CFU mL^−1^) was mixed with the nanocarrier sample and incubated for 3 h. After the incubation, the bacteria were collected by centrifugation, the supernatant was discarded, and electron microscopy fixative was added for fixation. Next, perform gradient ethanol dehydration according to conventional electron microscopy sample preparation procedures, and resuspend the sample in absolute ethanol. Take 100 μL of the resuspension and add it dropwise to the silicon wafer. After drying, sputter-plate with gold. Finally, use a ZEISS GeminiSEM300 scanning electron microscope to observe the bacteria.

Assess bacterial survival using the live/dead staining method. Stain live bacteria with DMAO (green) and dead bacteria with EthD-III (red). Add 1 ml treated bacterial suspension to a 1.5 ml tube, followed by 1 μl DMAO and 2 μl EthD-III dyes. Incubate in the dark for 15 min. Place 10 μl of bacterial suspension on a glass slide and capture images of live and dead bacteria using a confocal laser microscope.

### 2.26 Adoptive transfer experiment

To verify the function of TGFβ1^+^CCR5^+^ double-positive neutrophils in animal models, we established a mouse fracture model and divided mice into two groups (n = 6). We transplanted TGFβ1-CCR5-double-negative neutrophils in the control group and TGFβ1^+^CCR5^+^ double-positive neutrophils in the experimental group (CRS-Neu group). We transplanted cells on days 0, 7, 14, and 21, and transplanted 2.0 × 10^5^ cells each time. We took X-rays on days 0, 7, 14, and 21, and sacrificed animals on days 14 and 21. We performed micro-CT analysis and quantified BV/TV and BMD. After fixing the mouse fracture samples in paraformaldehyde, we performed HE and Masson staining to assess fracture healing.

### 2.27 Statistical analysis

All in vivo experiments were conducted under standardized randomization and blinding protocols to minimize selection bias and measurement bias. Data processing and statistical analysis were completed by researchers blinded to experimental grouping. All statistical analyses were performed using GraphPad Prism 9.0. All quantitative data were presented as mean ± standard deviation (SD). Before parametric tests, the Shapiro-Wilk test was applied to assess normality of each dataset, and Levene's test was used to verify homogeneity of variances. All in vitro experiments were independently repeated with at least 3 biological replicates; in vivo animal experiments, n = 6; biocompatibility test, n = 3. P < 0.05 was defined as statistically significant. Significance thresholds: ns P > 0.05, *P < 0.05, **P < 0.01, ***P < 0.001, ****P < 0.0001.

For comparisons between two groups: independent Student's t-test was used when data passed normality and equal variance tests; Welch's corrected *t*-test was adopted for normal data with unequal variances; the Mann-Whitney *U* test (nonparametric) was utilized for non-normal distributed data. For comparisons among three or more groups with normal distribution and equal variances: one-way analysis of variance (one-way ANOVA) followed by Tukey's HSD post-hoc test for all pairwise multiple comparisons. For datasets with normal distribution but unequal variances, Welch-corrected one-way ANOVA with Tamhane's T2 post-hoc test was performed. Non-normal multi-group datasets were analyzed via the Kruskal-Wallis H test, followed by Dunn's post-hoc test with P-value correction for multiple comparisons. Repeated-measures one-way ANOVA with Tukey's HSD post-hoc test was used for longitudinal time-series measurements (e.g., sequential X-ray observation, gradient time-point live/dead cell quantification).

## 3 Results

### 3.1 CRS impairs fracture healing and expands TGFβ1^+^CCR5^+^ neutrophils in mice

To investigate the effects of anxiety on fracture healing and neutrophil function, we exposed mice to 14 days of chronic restraint stress (CRS) to establish an anxiety model (Fig. S1A–B) [[40], [41], [42]]. CRS-exposed mice spent significantly less time in the open arms of the elevated plus maze and exhibited reduced central zone exploration with a peripheral preference in the open field test, confirming successful induction of anxiety-like behaviours (Fig. S1C and D). During the fracture healing period, mice were not subjected to further restraint to avoid any mechanical interference from immobilization that could confound bone repair assessment [43,44]. Nevertheless, dynamic behavioral monitoring demonstrated that anxiety-like behaviours persisted for at least 21 days after cessation of restraint (Fig. S1K and L). During 14 days of restraint stress, the plasma levels of corticosterone and norepinephrine in mice increased significantly and could continue to be maintained for a long time (Fig. S1E and F). The weight changes of mice during 14 days were also consistent with changes in anxiety stress (Fig. S1G). In addition, during the entire 21-day experiment, there were no significant differences in FST/TST immobility time and sugar water preference between CRS and control mice. This indicates that the mice did not exhibit despair behavior or pleasure deprivation throughout the experimental period, thus largely ruling out the existence of a depressive-like state and confirming the specificity of the anxiety model (Fig. S1H–J) [45,46].

Control and CRS mice were then subjected to femoral fracture surgery. X-ray radiographs revealed that CRS mice maintained clear fracture lines with minimal callus formation even at day 21 post-surgery, indicating markedly impaired healing (Fig. 1A). Micro-CT analysis further confirmed that callus density and volume, as well as BV/TV and bone mineral density (BMD), were significantly reduced in the CRS group (Fig. 1B). At the molecular level, both mRNA and protein levels of osteogenic markers, including *Col1a1*, *Bglap*, and *Runx2* were substantially downregulated in CRS mice (Fig. 1C and D). These results collectively indicate that CRS-induced anxiety strongly suppresses osteogenic activity and delays fracture healing.

**Fig. 1:**
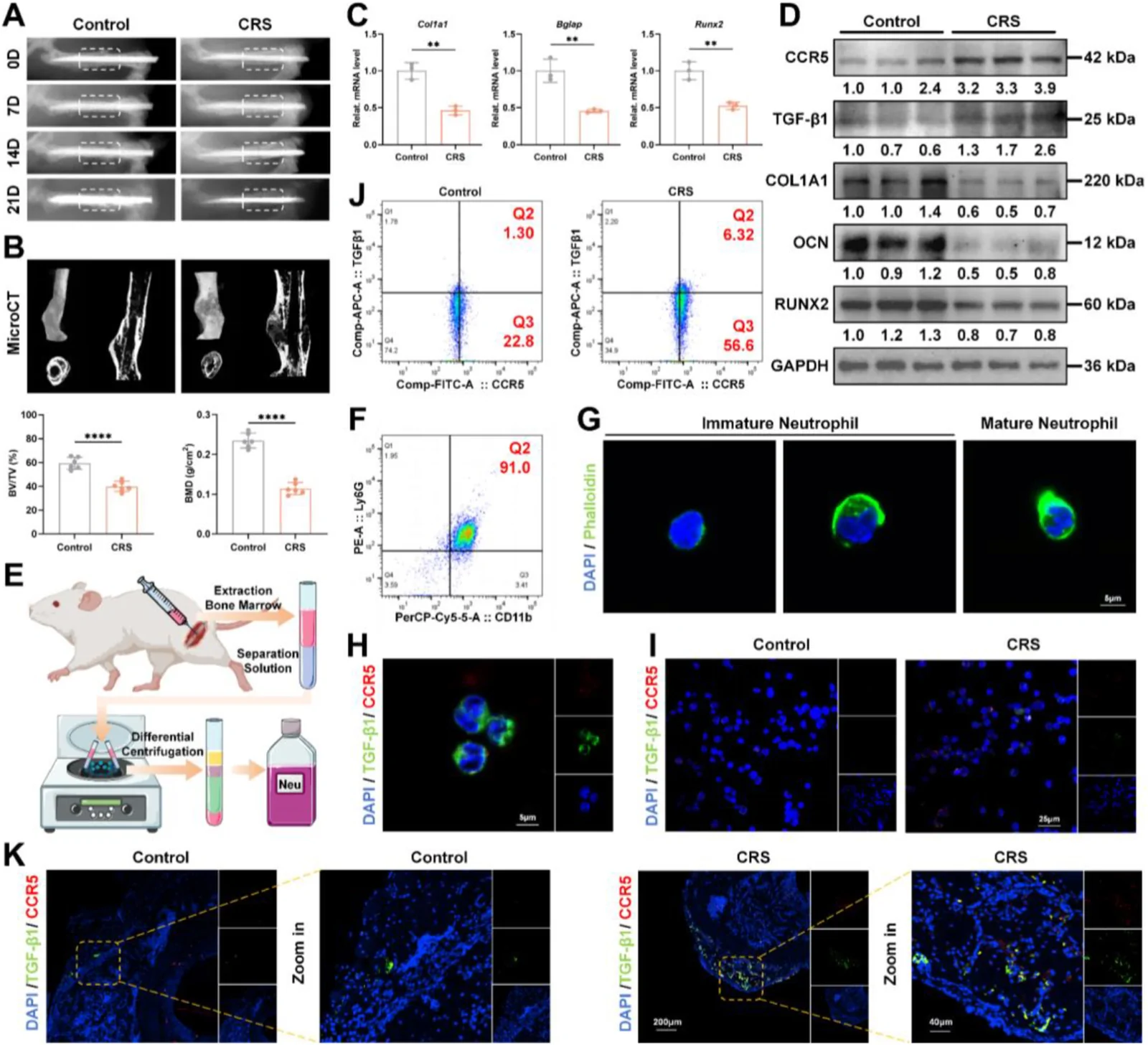
**CRS delays fracture healing and increases the proportion of TGFβ1^+^CCR5^+^ double-positive neutrophils in mice.** (A) X-ray imaging of the control and CRS groups on days 0, 7, 14, and 21 after fracture. n = 6. (B) Micro-CT three-dimensional reconstruction images of fracture ends and quantitative analysis of BV/TV and BMD in mice. n = 6. (C) mRNA levels of *Col1a1*, *Bglap*, and *Runx2*. n = 3. (D) Protein expression levels of CCR5, TGFβ1, and osteogenic markers (COL1A1, OCN, RUNX2). n = 3. (E) Schematic diagram of the isolation and extraction workflow for mouse primary neutrophils. (F) Phenotypic identification and purity verification of primary neutrophils by flow cytometry. (G) Morphological observation of primary neutrophils via immunofluorescence staining under laser confocal microscopy. Scale bar: 5 μm. (H) Morphology and subcellular localization of TGFβ1^+^CCR5^+^ double-positive neutrophils detected by immunofluorescence taining under laser confocal microscopy. Scale bar: 5 μm. (I) Representative images of immunofluorescence staining of TGFβ1^+^CCR5^+^ double-positive cells, scale bar: 25 μm. n = 3. (J) Flow cytometry shows the proportion of double-positive cells. n = 3. (K) Immunofluorescence images showing TGFβ1^+^CCR5^+^ double-positive cells at the site of bone callus in the control and anxiety-induced fracture groups. Scale bars: 200 μm, 40 μm.

Given that neutrophils are key effectors of early inflammatory responses during fracture and that inflammatory dysregulation is often an initiating event in bone repair impairment, we next examined the impact of CRS on bone marrow neutrophil phenotypes. Primary neutrophils were isolated by density gradient centrifugation (Fig. 1E), and flow cytometry confirmed that the isolated Ly6G^+^CD11b^+^ population reached 91.0% purity (Fig. 1F). Phalloidin and DAPI staining revealed characteristic segmented (mature) and band-shaped (immature) nuclear morphologies (Fig. 1G). High-magnification imaging showed the subcellular distribution of CCR5 and TGFβ1 within neutrophils (Fig. 1H). Notably, the proportion of TGFβ1^+^CCR5^+^ double-positive neutrophils was significantly higher in the CRS group than in controls (Fig. 1I), a finding consistently validated by both flow cytometry and immunoblotting (Fig. 1D–J). In addition to mouse bone marrow cells, we performed immunofluorescence staining on fracture sites in mice (Fig. 1K); the results were consistent with those for bone marrow cells, with the proportion of TGFβ1^+^CCR5^+^ double-positive cells being significantly higher in the anxiety group than in the control group. These results suggest that anxiety may initiate early abnormalities in the fracture healing microenvironment through specific expansion of this bone-related pathogenic neutrophil subset.

### 3.2 TGFβ1^+^CCR5^+^ neutrophils directly suppress BMSCs osteogenic differentiation in a ratio-dependent manner

We next sought to define the functional significance of TGFβ1^+^CCR5^+^ double-positive neutrophil expansion. The subset was enriched from mouse bone marrow neutrophils by MACS (Fig. 2A). Confocal microscopy confirmed co-expression of TGFβ1 and CCR5 in sorted cells and revealed their subcellular localization (Fig. 2B); flow cytometry determined the sorting purity to be 93.7%, which was considered sufficient for downstream functional assays (Fig. 2C).

**Fig. 2:**
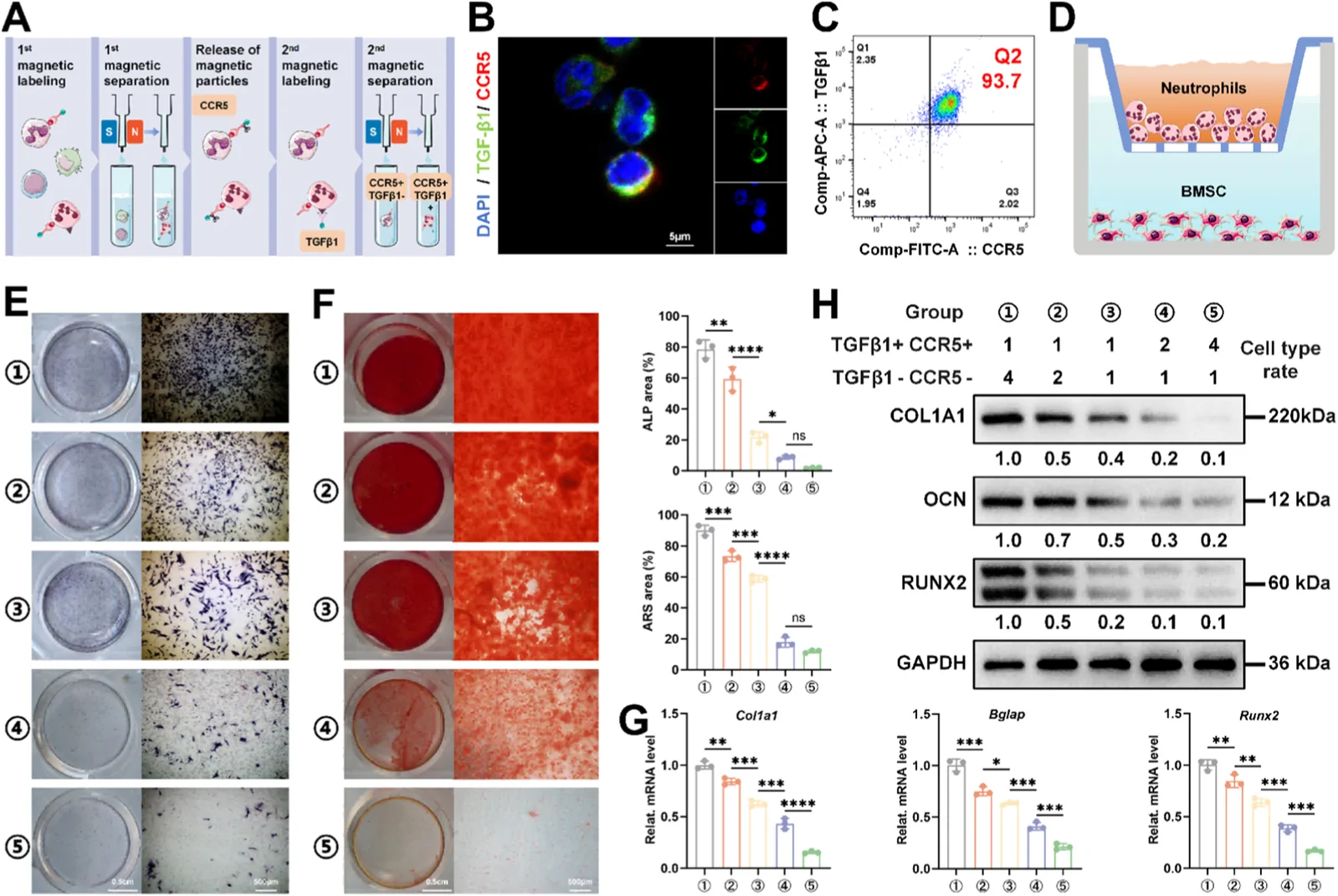
**TGFβ1^+^CCR5^+^ neutrophils inhibit osteogenic differentiation of BMSCs in co-culture.** (A) Schematic diagram of MACS for TGFβ1^+^CCR5^+^ double-positive neutrophils. (B) Immunofluorescence staining of TGFβ1 and CCR5 expression in sorted neutrophils. Scale bar: 5 μm. (C) The proportion of sorted double-positive cells was determined by flow cytometry. (D) Schematic representation of the Transwell co-culture system consisting of TGFβ1^+^CCR5^+^ neutrophils and BMSCs. (E) ALP staining and quantitative analysis of positive areas in BMSCs after 7 days of osteogenic induction in co-culture. n = 3. (F) Alizarin red staining and quantitative analysis of positive areas in BMSCs after 14 days of osteogenic induction in co-culture. n = 3. (G) mRNA levels of *Col1a1, Bglap, Runx2* in BMSCs following co-culture. n = 3. (H) Protein levels of COL1A1, OCN, and RUNX2 in BMSCs following co-culture. n = 3.

We then established a Transwell co-culture system in which BMSCs were cultured with graded mixtures of TGFβ1^+^CCR5^+^ and TGFβ1^−^CCR5^-^ neutrophils at ratios of 1:4, 1:2, 1:1, 2:1, and 4:1 (Fig. 2D). Increasing the proportion of double-positive neutrophils led to a stepwise reduction in BMSCs ALP activity (Fig. 2E) and diminished mineralized nodule formation (Fig. 2F). Consistently, mRNA levels of osteogenic marker genes *Col1a1*, *Bglap*, and *Runx2* (Fig. 2G), as well as protein levels of COL1A1, OCN, and RUNX2 (Fig. 2H), were progressively downregulated with increasing double-positive neutrophil ratios. Collectively, these data demonstrate that TGFβ1^+^CCR5^+^ double-positive neutrophils directly suppress BMSCs osteogenic differentiation in a ratio-dependent manner, implicating this subset as a functional driver of CRS-associated healing delay.

To validate the function of TGFβ1^+^CCR5^+^ double-positive neutrophils in an animal model, we established a mouse model of fracture and performed a transplant of TGFβ1^+^CCR5^+^ double-positive neutrophils (CRS-Neu group). The results showed that, compared with the group transplanted with an equal number of TGFβ1^−^CCR5^-^ double-negative neutrophils (control group), fracture healing was significantly delayed in mice transplanted with double-positive cells; on days 14 and 21, the fracture healing line was more pronounced (Fig. S2A). Micro-CT data revealed the same healing trend: in the double-positive cell transplantation group, the fracture ends were larger, the callus was disorganized, and the BV/TV and BMD values were lower (Fig. S2B). At day 21, HE and Masson's staining revealed that the fracture ends in the control group contained abundant new callus and chondral islands (Fig. S2C); whereas in the CRS-Neu group, the fracture ends were predominantly composed of fibrous connective tissue, with a clear fracture line and sparse chondrogenic formation. Masson's trichrome staining further confirmed that the callus region in the control group exhibited dense blue mature bone matrix, whilst the CRS-Neu group was dominated by red collagen fibers, with a significant reduction in blue bone area, suggesting that osteogenic activity was inhibited.

### 3.3 AKBA reduces the TGFβ1^+^CCR5^+^ neutrophil subset and rescues BMSCs osteogenic differentiation

Given that TGFβ1^+^CCR5^+^ double-positive neutrophils suppress BMSCs’ osteogenesis in a ratio-dependent manner, targeted reduction of this subset may represent an effective intervention. AKBA, one of the principal active components of frankincense resin, has demonstrated potent anti-inflammatory and immunomodulatory effects in inflammatory diseases such as rheumatoid arthritis, and its ability to modulate neutrophil function has also been reported. Based on these lines of evidence, AKBA was selected as the candidate drug for this study (chemical structure shown in Fig. 3A). We first determined the working concentration of AKBA in neutrophils using a CCK-8 assay; 10 μM was selected as it yielded optimal cell viability without significant toxicity (Fig. 3B). Live/dead staining further confirmed that this concentration caused no obvious cytotoxicity at 0, 6 or 12 h after treatment (Fig. 3C). Following 24 h of AKBA treatment, immunofluorescence staining revealed markedly reduced fluorescence intensities of CCR5 and TGFβ1, accompanied by a significant decrease in the proportion of TGFβ1^+^CCR5^+^ double-positive cells (Fig. 3D); flow cytometry confirmed this effect (Fig. 3E).

**Fig. 3:**
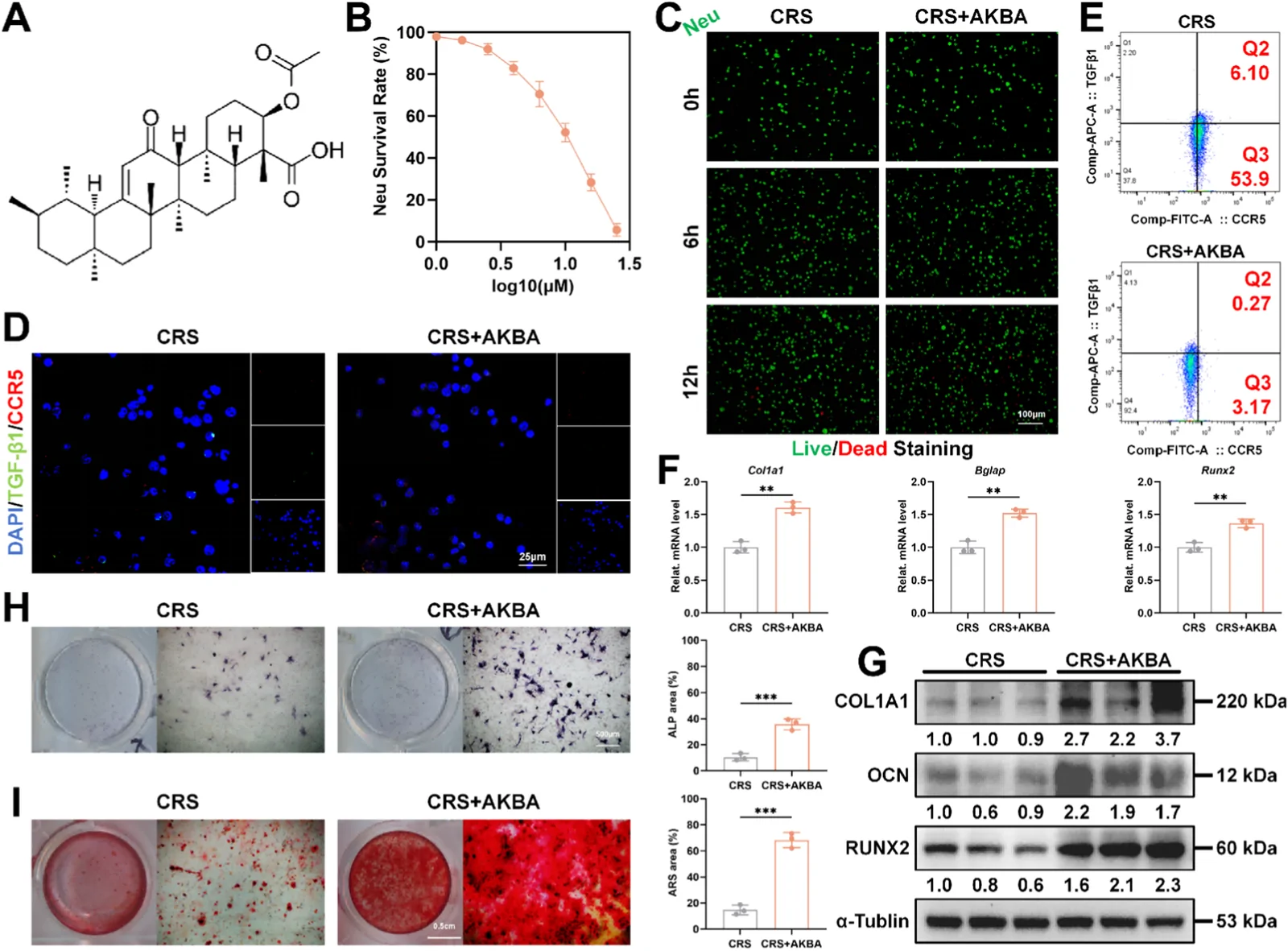
**AKBA suppresses the proportion of TGFβ1^+^CCR5^+^ neutrophils and rescues BMSCs osteogenic differentiation.** (A) Chemical structure of AKBA. (B) CCK-8 assay of neutrophil viability after 24 h of AKBA treatment at gradient concentrations. (C) Live/dead staining of neutrophils following AKBA treatment at 0, 6, and 12 h. (D) Immunofluorescence staining and quantification of TGFβ1^+^CCR5^+^ double-positive neutrophils after AKBA intervention. (E) The expression levels of TGFβ1 and CCR5 in neutrophils after AKBA treatment were analyzed by flow cytometry. (F) mRNA levels of *Col1a1, Bglap, and Runx2* in BMSCs co-cultured with AKBA-treated neutrophils. (G) Protein levels of COL1A1, OCN, and RUNX2 in BMSCs from the co-culture system. (H) ALP staining and quantitative analysis of BMSCs after 7 days in co-culture. (I) Alizarin red staining of BMSCs after 14 days in co-culture. n = 3.

Subsequently, AKBA-pretreated neutrophils were co-cultured with BMSCs. The mRNA levels of osteogenic marker genes *Col1a1, Bglap, and Runx2* (Fig. 3F), as well as the protein levels of COL1A1, OCN, and RUNX2 (Fig. 3G), were all significantly upregulated in BMSCs. Functionally, ALP activity was markedly enhanced at day 7 (Fig. 3H), and mineralized nodule formation was substantially increased at day 14 (Fig. 3I). Together, these results demonstrate that AKBA relieves the osteogenic suppression imposed by TGFβ1^+^CCR5^+^ neutrophils through reducing this pathogenic subset, thereby restoring bone-forming capacity.

### 3.4 AKBA regulates the USP31-NF-κB-CCR5 signaling axis by directly binding to YAP1 in neutrophils

To elucidate the molecular mechanism by which AKBA reduces the TGFβ1^+^CCR5^+^ neutrophil fraction, we performed transcriptomic sequencing on primary neutrophils from the CRS and AKBA-treated groups. The volcano plot showed that CCR5 transcription was significantly downregulated following AKBA treatment (Fig. 4A). KEGG enrichment analysis revealed marked inhibition of the NF-κB signaling pathway (Fig. 4B), and chord diagram analysis further confirmed NF-κB as a key upstream regulator of CCR5 expression (Fig. 4C). Given that YAP1 has been reported to enhance NF-κB activity through suppression of USP31 [47,48], we examined the nodal changes within this pathway. Compared with the control group, CRS significantly suppressed Usp31 transcription and elevated p-p65 protein levels; AKBA treatment reversed these effects, upregulating USP31 expression and reducing p-p65 abundance (Fig. 4D and E). Immunofluorescence staining revealed that NF-κB nuclear translocation was markedly enhanced in immature neutrophils from the CRS group, whereas AKBA treatment substantially attenuated this translocation (Fig. 4F). These results indicate that AKBA downregulates CCR5 expression by inhibiting NF-κB phosphorylation and nuclear translocation via the YAP1 protein.

**Fig. 4:**
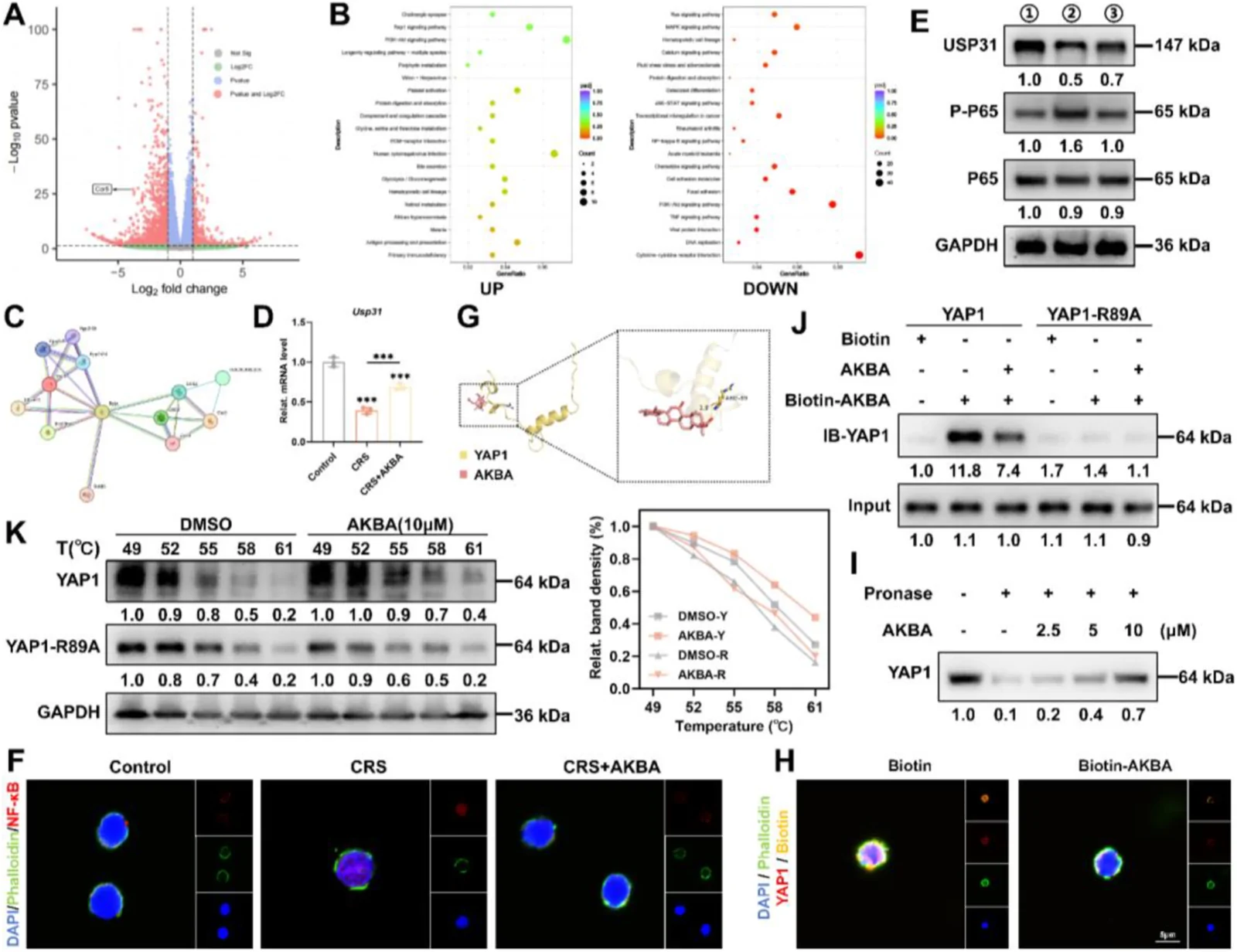
**AKBA exerts its effects by regulating the NF-κB-CCR5 axis and targeting YAP1 in neutrophils.** (A) Volcano plot of transcriptomic sequencing in neutrophils. (B) KEGG pathway enrichment analysis. (C) Chord plot analysis of key genes and pathways regulating CCR5 expression. (D) mRNA levels of NF-κB-CCR5 axis-related molecules in the control, CRS, and CRS + AKBA groups. n = 3. (E) Protein levels of NF-κB-CCR5 axis-related molecules in the control, CRS, and CRS + AKBA groups. n = 3. (F) Immunofluorescence staining showing that AKBA treatment attenuates NF-κB nuclear translocation in neutrophils. n = 3. (G) Molecular docking analysis of the binding mode, key interacting residues, and binding free energy between AKBA and YAP1 using Autodock Vina. (H) Intracellular co-localization of FITC-AKBA and YAP1 observed by confocal laser microscopy. (I) Binding of AKBA to YAP1 confirmed by drug affinity responsive target stability assay (DARTS). (J) Biotin pull-down assays in Hoxb8 cells confirmed the direct binding of biotin-AKBA bound YAP1 but not the YAP1-R89A mutant. (K) The thermal stability of YAP1 and YAP1-R89A after AKBA treatment was assessed by Cellular Thermal Shift Assay in Hoxb8 cells.

To identify AKBA's direct target, we performed molecular docking experiments in vitro. The results showed that the binding free energy between AKBA and YAP1 was −7.8 kcal/mol, indicating a strong binding affinity between the two (Fig. 4G). Immunofluorescence colocalization experiments demonstrated significant spatial overlap between AKBA and YAP1 within cells (Fig. 4H). Proteolytic degradation assays confirmed that AKBA inhibited pronase-induced YAP1 degradation in a concentration-dependent manner (Fig. 4I). We performed 100 ns all-atom molecular dynamics simulations to comprehensively analyze the conformational dynamics, structural stability, intermolecular interactions, and thermodynamic properties of the AKBA–YAP1 complex. As shown in Fig. S3A–G, the root-mean-square deviation (RMSD) curve stabilized following initial conformational adjustment, the radius of gyration (Rg) decreased significantly from 1.9 nm to 1.2 nm (indicating stable folding), and the free energy landscape (FEL) exhibited the characteristics of a single deep potential well corresponding to a thermodynamically stable ground-state conformation. These analyses confirmed a stable binding between AKBA and YAP1.

In order to verify that the AKBA-YAP1 target interaction also exists in primary neutrophils collected from CRS animals at the fracture site, we created a CRS fracture animal model and treated the animal model with AKBA at a concentration of 5 mg/kg. We sacrificed the mice 12 h later and extracted neutrophils from the fracture site as described in the methodology section. We performed immunofluorescence colocalization experiments on primary neutrophils collected from fracture sites, which showed significant spatial overlap between intracellular AKBA and YAP1 (Fig. S4A). We treated mice with AKBA at 0 mg/kg, 1.25 mg/kg, 2.5 mg/kg, and 5 mg/kg, isolated neutrophils, and performed proteolytic degradation assays. The results confirmed that AKBA inhibited pronase-induced YAP1 degradation in a concentration-dependent manner (Fig. S4B). This result increases tissue-level evidence that AKBA and YAP1 interact.

To better study neutrophil gene expression, we introduced Hoxb8 cells. Hoxb8 cells are conditionally immortalized mouse bone marrow progenitor cells commonly used for in vitro neutrophil studies. We achieved gene knockdown by siRNA transfection at the progenitor stage, then induced differentiation by removing estrogen and supplementing granulocyte colony-stimulating factor (G-CSF) to generate neutrophils, bypassing the difficulty of transfecting terminally differentiated cells [49,50].We first treated Hoxb8 cells with 0, 5, 10, 15 μM AKBA and used live-dead staining to detect cell viability. We observed that 10 μM AKBA did not affect Hoxb8 cells (Fig. S4C). In order to further confirm that YAP1 is the target of AKBA, we used siRNA to knock down YAP1 in Hoxb8 cells and induced maturation to observe whether the effects caused by excess free AKBA (15 μM) would be weakened with YAP1 knockdown. We performed PCR (Fig. S4D) and WB (Fig. S4E) to detect downstream USP31 and found that AKBA increased USP31 transcription and expression, while YAP1 knockdown weakened this effect. WB (Fig. S4E) and immunofluorescence experiments (Fig. S4F) confirmed that AKBA reduced the phosphorylation ratio and nuclear translocation of P65, while YAP1 knockdown weakened this effect.

Given that the AKBA molecule forms a critical interaction with arginine at position 89 (R89) on the surface of the YAP1 protein (Fig. 4G), we used Hoxb8 cells for subsequent experiments. We introduced point mutations into YAP1 to generate the YAP1-R89A mutant and induced differentiation into neutrophils. Biotin pull-down assays (Fig. 4J) confirmed that AKBA could precipitate YAP1 protein from cell lysates, but failed to precipitate the YAP1-R89A mutant. Cellular Thermal Shift Assay (CETSA) (Fig. 4K) showed that AKBA treatment significantly increased the thermal stability of YAP1, but did not enhance the stability of the YAP1-R89A mutant. These results confirm the interaction between YAP1 and AKBA, and identify arginine at position 89 on the YAP1 surface as the key interaction site. In summary, AKBA directly binds to YAP1, relieves YAP1-mediated suppression of USP31, thereby blocking NF-κB phosphorylation and nuclear translocation, and ultimately downregulates CCR5 expression and reduces the TGFβ1^+^CCR5^+^ pathogenic neutrophil subset.

### 3.5 Functional necessity of the YAP1–USP31–NF-κB–CCR5 axis is validated in Hoxb8 and HL-60 cells

In order to verify that YAP1–USP31–NF-κB regulates the expression of CCR5, we further performed gene expression intervention in Hoxb8 cells and observed changes in downstream signaling pathways. Our experiments were divided into four groups: Hoxb8, Hoxb8+AKBA, Hoxb8+AKBA + YAP1 overexpression and Hoxb8+AKBA + USP31 silencing. In Hoxb8 cells, AKBA treatment significantly upregulated USP31 transcription and downregulated CCR5 transcription; these effects were reversed by either YAP1 overexpression or USP31 silencing (Fig. 5B). At the protein level, AKBA increased USP31 expression, suppressed p-p65 phosphorylation, and reduced CCR5 protein abundance; YAP1 overexpression or USP31 silencing abrogated these changes (Fig. 5A). In parallel, AKBA treatment markedly attenuated NF-κB nuclear translocation [51]and reduced the proportion of TGFβ1^+^CCR5^+^ double-positive cells, and these effects were similarly reversed by YAP1 overexpression or USP31 silencing (Fig. 5C and D).

**Fig. 5:**
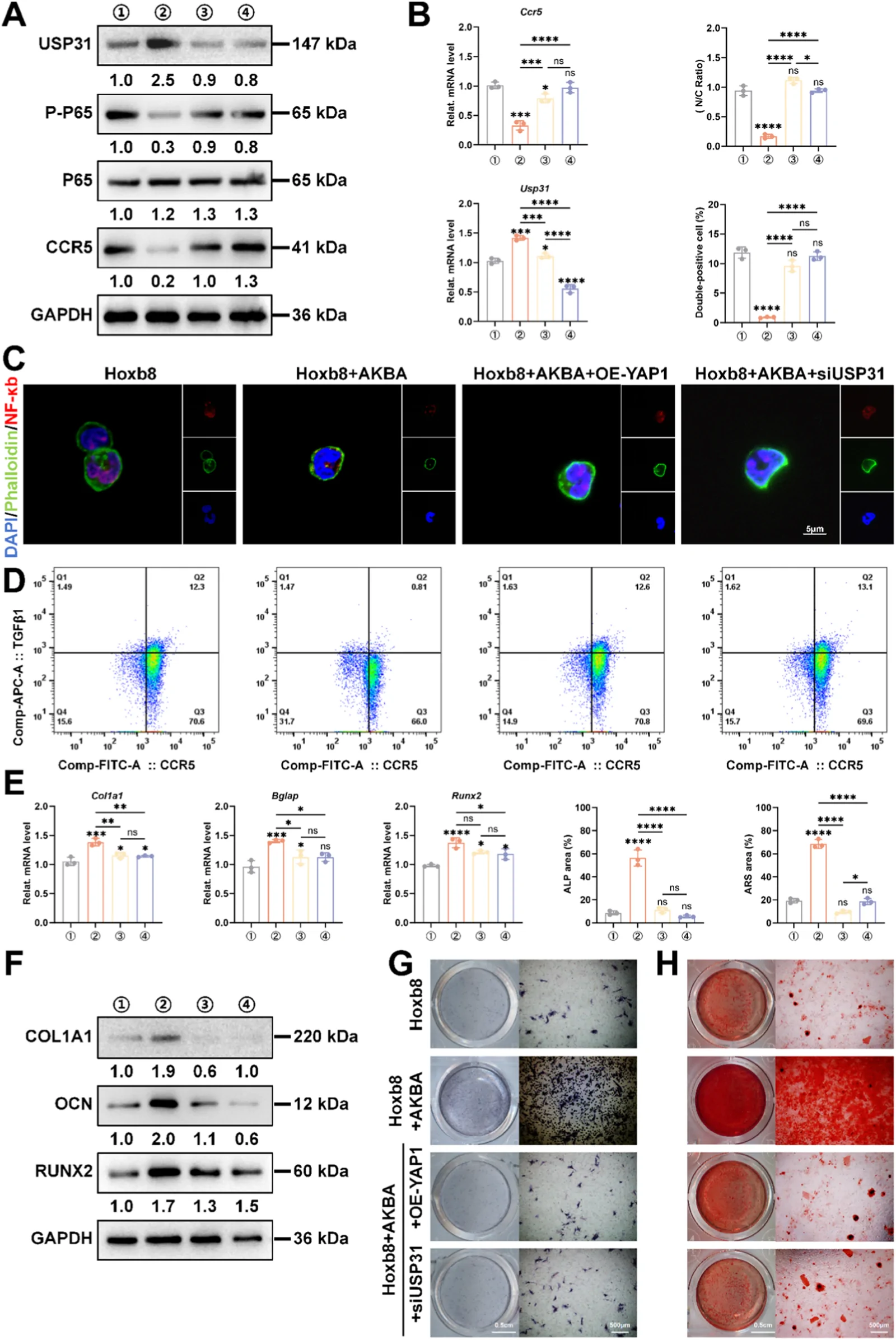
**AKBA exerts its effects by regulating the YAP1-USP31-NF-κB-CCR5 axis in Hoxb8 cells.** Four groups were included: Hoxb8, Hoxb8+AKBA, Hoxb8+AKBA + OE-YAP1, and Hoxb8+AKBA + si USP31. (A) Protein levels of USP31, NF-κB, and CCR5 in Hoxb8 cells. n = 3. (B) mRNA levels of Usp31 and Ccr5 in Hoxb8 cells. n = 3. (C) NF-κB nuclear translocation in Hoxb8 cells observed by confocal laser microscopy, and analysis of NF-κB nuclear-cytoplasmic fluorescence ratio. n = 3. (D) The proportion of CCR5^+^ and TGFβ1^+^ Hoxb8 cells was analyzed by flow cytometry and double-positive cell ratio statistics. n = 3. (E) mRNA levels of osteogenic genes (Col1a1, Bglap, Runx2) in BMSCs from co-culture systems. n = 3. (F) Protein levels of osteogenic markers (COL1A1, OCN, RUNX2) in BMSCs from co-culture systems. n = 3. (G) Osteogenic activity of BMSCs in co-culture systems assessed by ALP staining. n = 3. (H) Mineralized nodule formation of BMSCs in co-culture systems was evaluated by Alizarin Red staining. n = 3.

To further validate the regulatory role of this signaling axis in BMSCs osteogenic differentiation, Hoxb8 cells from each group were co-cultured with BMSCs. AKBA-treated Hoxb8 cells significantly upregulated the mRNA levels of osteogenic marker genes *Col1a1*, *Bglap*, and *Runx2* (Fig. 5E) as well as the protein levels of COL1A1, OCN, and RUNX2 (Fig. 5F), and enhanced ALP activity (Fig. 5G) and mineralized nodule formation (Fig. 5H). These pro-osteogenic effects were abrogated by YAP1 overexpression or USP31 silencing. Collectively, these results confirm that AKBA regulates CCR5 expression and reduces the TGFβ1^+^CCR5^+^ pathogenic subset through the YAP1–USP31–NF-κB–CCR5 axis, thereby relieving BMSCs osteogenic suppression, and that this regulatory pathway is functionally required.

To further rule out potential species-specific effects and improve the translational relevance of the results, we expanded our preliminary observations from the mouse cell line to the human cell line HL-60. In HL-60 cells, we performed the same experiment and first confirmed the safety of 10 μmol/L AKBA by live/dead staining (Fig. S5A). In HL-60 cells, AKBA treatment significantly upregulated USP31 transcription and downregulated CCR5 transcription, while YAP1 overexpression or USP31 silencing reversed these changes (Fig. S5B). At the protein level, AKBA increased USP31 expression, inhibited p-p65 phosphorylation, and reduced CCR5 protein abundance. YAP1 overexpression or USP31 silencing abolished these effects (Fig. S5C). In addition, AKBA treatment significantly attenuated NF-κB nuclear translocation and reduced the proportion of TGFβ1^+^CCR5^+^ double-positive cells, and YAP1 overexpression or USP31 silencing also reversed these effects (Fig. S5D and E). Co-culture experiments with BMSCs also further supported this finding. In HL-60 cells treated with AKBA, the mRNA levels of osteogenic marker genes Col1a1, Bglap, and Runx2 (Fig. S5F) and the protein levels of COL1A1, OCN, and RUNX2 (Fig. S5G) were significantly increased, and ALP activity (Fig. S5H) and mineralized nodule formation (Fig. S5I) were also enhanced; YAP1 overexpression or USP31 silencing could eliminate these bone-promoting effects. These HL-60 data suggest potential cross-species relevance of this regulatory axis, but confirmation in primary human neutrophils is needed before claiming human conservation.

Previous studies reported that TGFβ1^+^CCR5^+^ neutrophils secrete TGF-β1 to activate the TRAF3-NF-κB-CCL5 pathway in BMSCs and form an inflammatory amplification loop [23]. We therefore performed co-culture experiments using neutrophils and BMSCs from the control and anxiety groups. The results indicated that CRS induces CCL5 expression by activating the TRAF3/NF-κB axis, but this effect is attenuated by AKBA and TGF-β antagonism (Fig. S6A–C). We subsequently validated these findings by subjecting the BMSCs from the co-culture system to osteogenic differentiation. We found that BMSCs co-cultured with anxiety-induced neutrophils exhibited reduced ALP activity and a decrease in the area of Alizarin Red-positive regions; this effect was inhibited by AKBA and TGF-β antagonism (Fig. S6D and E). These multi-dimensional assays confirmed abnormal activation of the TRAF3-NF-κB-CCL5 pathway in BMSCs in the CRS-related fracture model. To investigate whether the release of CCL5 by inflamed BMSCs might in turn interfere with neutrophil function, we first co-cultured BMSCs with both unstimulated neutrophils and AKBA-treated neutrophils, and then used a Transwell assay to assess the chemotactic effect of BMSCs on neutrophils. The results showed that BMSCs treated with anxiety-induced neutrophils exhibited a significant chemotactic effect on neutrophils, with an increase in the number of neutrophils crossing the membrane; however, following CCL5 neutralization, the number of neutrophils crossing the membrane decreased. When BMSCs were treated with AKBA-treated neutrophils, the chemotactic effect of the BMSCs on neutrophils was attenuated, but this effect was restored upon supplementation with exogenous CCL5 (Fig. S6F). To verify that neutrophil CCR5 is also involved in the inflammatory amplification circuit, we performed CCR5 knockdown and overexpression in Hoxb8 cells. The results showed that anxious BMSC significantly promoted Hoxb8 cells migration, while CCR5 knockdown reduced the number of migrating cells. After AKBA treatment, the migration rate decreased, but CCR5 overexpression increased Hoxb8 cells migration again (Fig. S6G). This result confirms the existence of an inflammatory amplification loop.

### 3.6 A neutrophil-targeted EGCG-coated nanocarrier enables delivery and release of AKBA

To achieve greater neutrophil enrichment with AKBA and minimize off-target effects, we constructed an NIMP-R14 aptamer-modified nanocarrier system with an epigallocatechin gallate (EGCG) shell (AKBA@E-N, preparation workflow shown in Fig. 6A). We analyzed the XPS spectra (Fig. S7A), Raman spectra (Fig. S7B), and FTIR spectra (Fig. S7C) of EGCG, AKBA@E and AKBA@E-N. EGCG self-polymerizes into uniform coatings under alkaline conditions and effectively loads AKBA [[52], [53], [54], [55]], while the NIMP-R14 aptamer mediates specific recognition of neutrophils [[56], [57], [58], [59]]. Transmission electron microscopy revealed that AKBA nanoparticles were spherical in shape with an intact EGCG coating on the surface (Fig. 6B). Dynamic light scattering measurements of the average particle size and Zeta potential of AKBA@E-N are shown in Fig. 6C and D. An ultra-fine pH microelectrode is inserted directly into the fracture haematoma or callus and connected to a pH meter for real-time readings. This is the most classic and direct method, enabling in vivo multi-point measurements [60,61]. We measured the pH at the fracture healing site and found that by the fourth day, the pH had dropped to as low as 6.69–6.89 (Fig. S7D) [62,63], which promotes EGCG coating degradation, we examined the release behaviour of AKBA@E-N at different pH values and confirmed that low pH accelerated EGCG release (Fig. 6E). We used liquid chromatography–tandem mass spectrometry (LC–MS/MS) to measure the concentration of the drug AKBA and obtained a standard calibration curve (Fig. S7E). Based on the measurements, we calculated the encapsulation efficiency (EE%) to be 83.5% ± 2.1% and the drug loading content (LC%) to be 13.2% ± 0.4%.

**Fig. 6:**
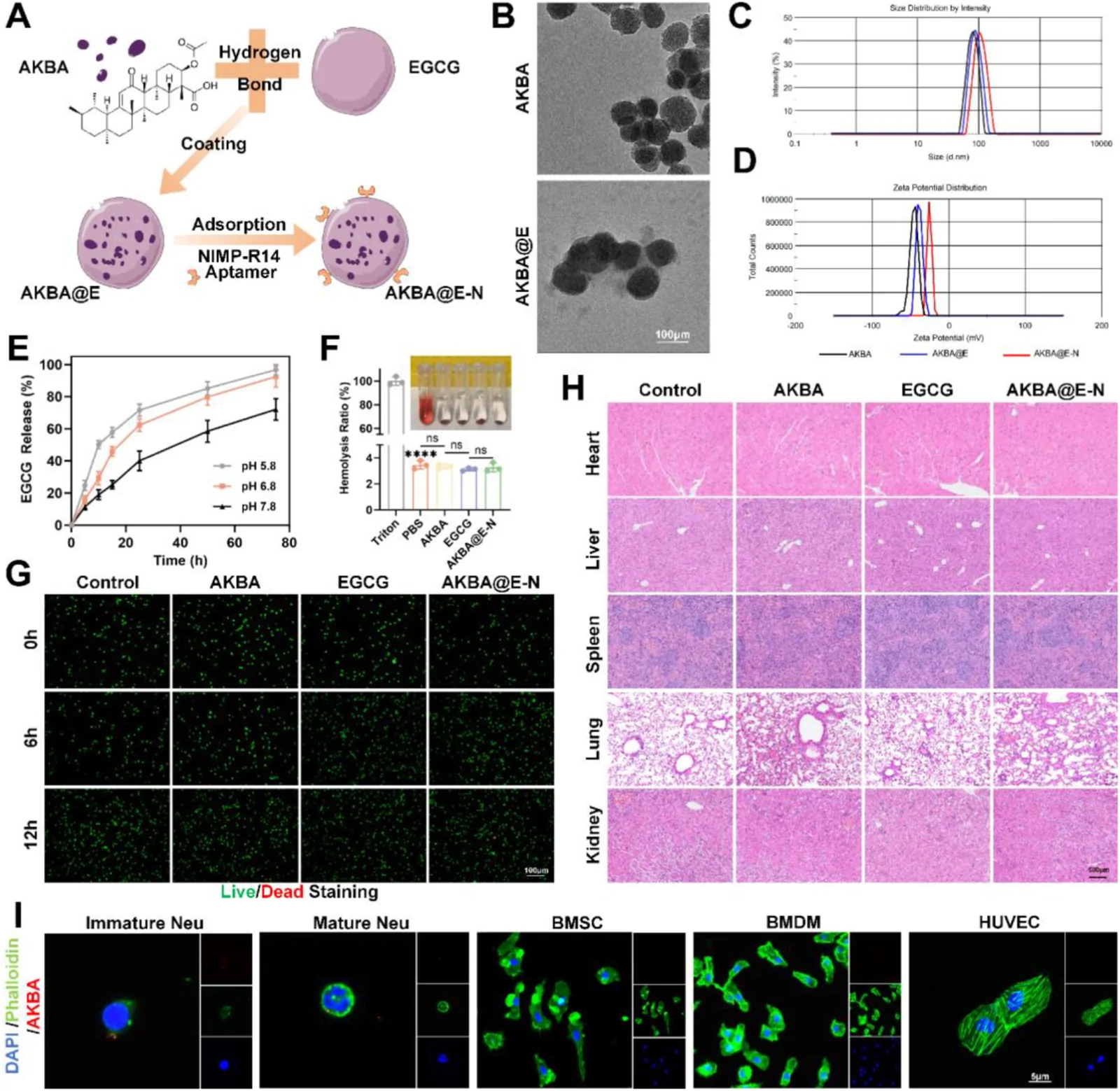
**Neutrophil-targeted coated material enables delivery and release of AKBA.** (A) Schematic illustration of the synthesis workflow for AKBA@E-N. (B) Micro-morphology of AKBA nanoparticles and EGCG-coated AKBA observed by transmission electron microscopy (TEM). (C) Particle size distribution of materials in different groups for the evaluation of size uniformity. (D) Zeta potential of materials in different groups for the Characterisation of surface charge properties. (E) Release behavior of AKBA@E-N at different pH values. (F) In vitro hemolysis assay of each group. n = 3. (G) Live/dead staining of neutrophils in each group. n = 3. (H) HE staining of the mouse heart, liver, spleen, lung, and kidney. n = 3. (I) Specific phagocytosis of fluorescent-labeled, coated, drug-loaded materials by neutrophils, BMSCs, BMDMs, and HUVECs, detected by confocal laser microscopy. n = 3.

We next evaluated the vector for enhanced neutrophil uptake. Cy3-labeled AKBA@E-N was co-incubated with mature neutrophils, immature neutrophils, BMSCs, bone marrow-derived macrophages (BMDMs) and human umbilical vein endothelial cells (HUVECs). Confocal microscopy showed that only mature and immature neutrophils exhibited efficient fluorescent uptake, whereas no appreciable phagocytosis was observed in other cell types (Fig. 6I), confirming the selective recognition capacity of the carrier for neutrophils. We then proceeded to validate the findings in an animal model. Following administration of AKBA, AKBA@E and AKBA@E-N to mice with fractures, we measured drug concentrations at the fracture site (Fig. S7F), and serum drug concentrations (Fig. S7G) at 72 h. AKBA@E-N exhibited a lower peak drug concentration, indicating improved drug safety; a delayed time to peak concentration, indicating sustained-release of the drug; the area under the concentration-time curve revealed higher total drug content at the fracture site and lower drug content in serum, suggesting better retention of the drug at the fracture site. We labeled neutrophils with LY6G and AKBA with Cy3, and performed immunofluorescence analysis of the fracture healing site (Fig. S7H); the AKBA@E-N group showed greater fluorescence overlap between the drug and LY6G, indicating that the drug is effectively enriched in neutrophils. We used flow cytometry to measure drug fluorescence intensity in neutrophils. We used FITC-labeled drug AKBA and flow cytometry to detect drug phagocytosis in bone marrow neutrophils. We first circled double-positive neutrophils using the ly6g and cd11b gates (Q2 gate) (Fig. S7I). We analyzed average fluorescence intensity in double-positive neutrophils, as shown in Fig. S7J. The average fluorescence intensity of the AKBA@E-N group was 14070, higher than 11646 for AKBA@E and 8471 for the AKBA group, indicating that the AKBA@E-N group contained more drugs. We imaged the viscera and the fracture site 24 h after material administration to observe and quantitatively analyze drug concentrations at various sites (Fig. S7K). It was observed that the drug in the AKBA@E-N group accumulated more effectively at the fracture site, whilst the lowest drug concentration was found in the liver, where the drug is metabolized; these findings successfully confirmed the selective accumulation of the drug at the fracture site or within neutrophils.

Biocompatibility assessment demonstrated that AKBA@E-N did not induce hemolysis (Fig. 6F), exerted no significant cytotoxicity towards neutrophils (Fig. 6G), and caused no pathological damage in major organs as assessed by HE staining (Fig. 6H). In addition, antibacterial assays in the supplementary materials indicated that the EGCG shell endowed AKBA@E-N with favorable in vitro antibacterial activity (Fig. S8A–F).

### 3.7 Verify the therapeutic effect of AKBA@E-N in vivo

On this basis, we evaluated the in vivo therapeutic efficacy of AKBA@E-N in the CRS-induced anxiety fracture mouse model (Fig. 7A). Mice were divided into seven groups: Control (normal fracture without CRS), CRS, CRS + AKBA, CRS + EGCG, CRS+@E-N, CRS + AKBA@E, and CRS + AKBA@E-N, with local administration at the fracture site. Longitudinal X-ray monitoring revealed that fracture lines in the AKBA@E-N group became markedly blurred from day 14 and approached complete healing by day 21, whereas those in the CRS group remained clearly visible; the AKBA group showed intermediate improvement (Fig. 7B). Micro-CT quantitative analysis confirmed that BV/TV and BMD in the AKBA@E-N group were significantly higher than those in all other treatment groups (Fig. 7C). Histological staining showed that the AKBA@E-N group exhibited well-organized callus architecture and a higher proportion of newly formed bone (HE staining), as well as the largest and most intensely stained red areas on Masson's trichrome staining, indicating the highest degree of bone maturity (Fig. 7D). Collectively, these results indicate that AKBA@E-N significantly enhances the in vivo efficacy of AKBA through selective enrichment of neutrophils and effectively rescues CRS-related fracture healing impairment.

**Fig. 7:**
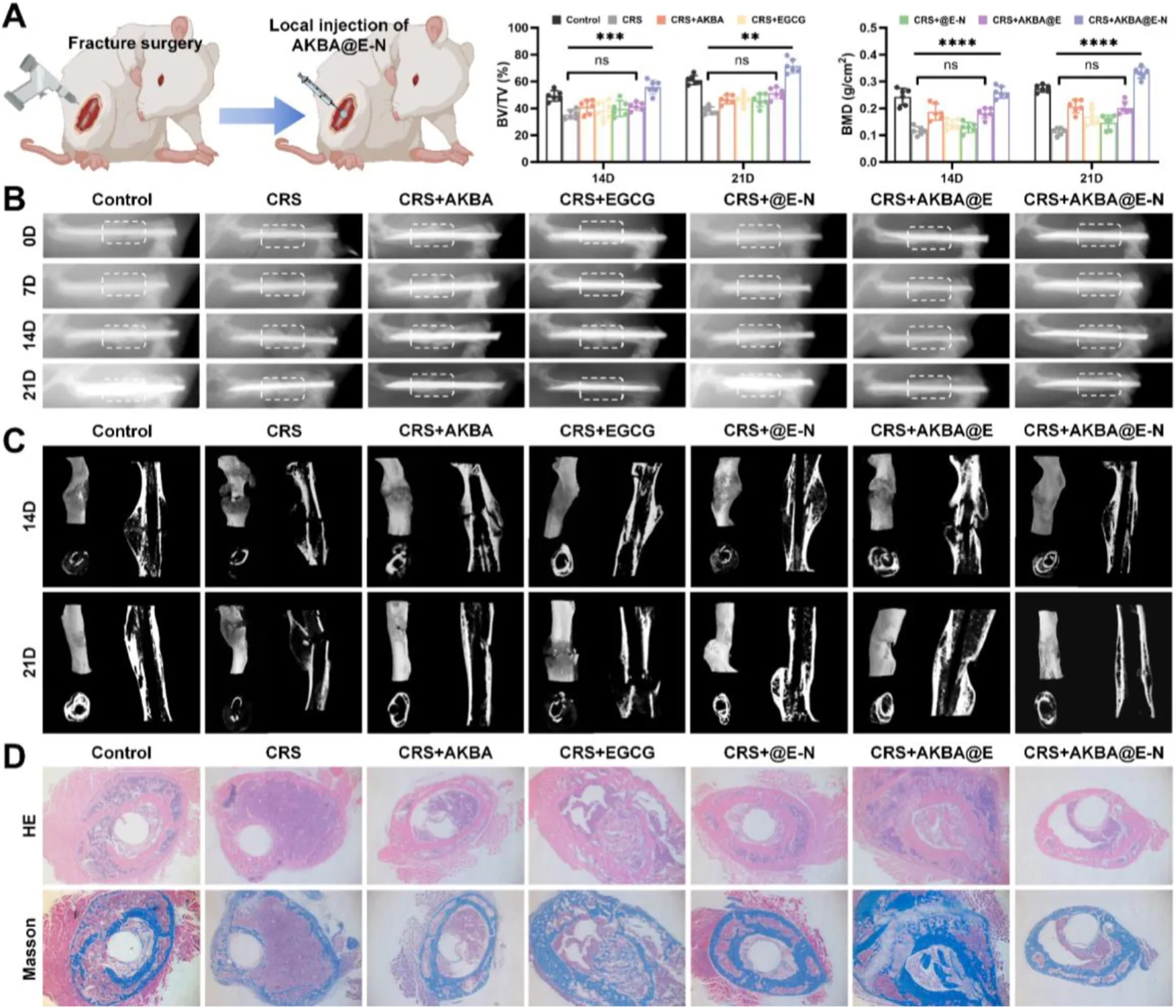
**Therapeutic efficacy of AKBA@E-N in mice with anxiety-related fracture.** (A) Schematic illustration of the in vivo intervention workflow and mechanism of AKBA@E-N in anxiety-related fracture mice. Mice were divided into seven groups: Control, CRS, CRS + AKBA, CRS + EGCG, CRS+@E-N, CRS + AKBA@E, and CRS + AKBA@E-N. n = 6. (B) X-ray images at 0, 7, 14, and 21 days after fracture were evaluated to evaluate the macroscopic process of fracture healing in each group. n = 6. (C) Micro-CT three-dimensional reconstruction images of the fracture callus, along with quantitative analysis of BMD and BV/TV to assess callus formation and bone repair. n = 6. (D) Masson's trichrome and HE staining of fracture callus tissues to evaluate collagen deposition, bone maturity, callus morphology, new bone distribution, and cell infiltration in each group. n = 6.

## 4 Discussion

The present study systematically elucidates the mechanism by which anxiety delays fracture healing through expansion of the TGFβ1^+^CCR5^+^ pathogenic neutrophil subset and proposes a therapeutic strategy combining AKBA with targeted delivery. The key findings are as follows: CRS-induced anxiety significantly increased the proportion of TGFβ1^+^CCR5^+^ double-positive neutrophils in the bone marrow, and this subset directly suppressed BMSCs' osteogenic differentiation in a ratio-dependent manner. AKBA directly binds to YAP1, relieves YAP1-mediated suppression of USP31, and consequently blocks NF-κB phosphorylation and nuclear translocation, leading to downregulation of CCR5 expression and reduction of the pathogenic subset. Building on this mechanistic insight, we developed an NIMP-R14-modified EGCG nanocarrier for local administration at the fracture site. This system enhanced neutrophil-preferential uptake and local retention of AKBA. It enabled fracture-microenvironment-responsive drug release, which significantly promoted bone healing in the CRS-induced anxiety fracture mouse model.

Notably, the TGFβ1^+^CCR5^+^ neutrophil-mediated pathogenic axis identified in this study is mechanistically and functionally distinct from the previously reported stress-induced bone injury pathway driven by tyrosine hydroxylase-positive (TH^+^) neutrophils. These two subsets act as parallel, independent regulatory branches, with core differences in upstream stress-sensing signals, cellular origin, downstream effector loops, and final skeletal phenotypes. For upstream activation, TH^+^ neutrophils rely entirely on the sympathetic nervous system-β_2_-adrenergic receptor axis: stress-elevated norepinephrine directly induces TH expression and endogenous catecholamine synthesis, and this phenotype is rapidly reversible after stress relief [64]. By contrast, the expansion of TGFβ1^+^CCR5^+^ neutrophils stems from intrinsic transcriptional reprogramming of bone marrow neutrophils via the YAP1–USP31–NF-κB–CCR5 cascade, independent of direct sympathetic innervation. This abnormal subset persists for at least 21 days after stress cessation, matching the chronic feature of clinical anxiety. Regarding cellular properties, TH^+^ neutrophils represent a transient activated state of circulating mature neutrophils recruited to fracture sites, and are not a bone marrow-resident population [64]. TGFβ1^+^CCR5^+^ neutrophils, however, are an innate bone marrow-resident subset that maintains physiological skeletal homeostasis, and expand in situ within the bone marrow niche under chronic anxiety [23]. At the downstream effector level, TH^+^ neutrophils exert unidirectional osteogenic inhibition via secreted catecholamines, with no reciprocal amplification loop with stromal cells. In sharp contrast, TGFβ1^+^CCR5^+^ neutrophils form a self-perpetuating positive feedback loop with BMSCs through the TGFβ1–TRAF3–NF-κB–CCL5 axis, which continuously exacerbates osteogenic suppression and constitutes a core pathological feature absent in the TH^+^ pathway [23]. These mechanistic differences translate into distinct repair outcomes. TH^+^ neutrophils mainly function in the early inflammatory phase (0–7 days) and cause mild, transient healing disturbance. The TGFβ1^+^CCR5^+^ subset exerts sustained inhibition throughout the entire repair process, peaking at the critical cartilage-to-bone transition window (14–21 days), leading to severe, persistent healing delay that better recapitulates the clinical phenotype of chronic anxiety-related bone impairment. Collectively, our study uncovers a previously unrecognized bone-specific innate immune regulatory axis for anxiety-related fracture delay, expanding the mechanistic framework of stress-skeletal interactions.

At the mechanistic level, this study defines the direct target and signaling pathway through which AKBA acts in fracture healing. Although AKBA has been reported to possess anti-neuroinflammatory, antioxidant, and osteogenic-promoting activities [28,30,65,66], its direct binding protein and upstream regulatory network have remained unclear. Through the combined application of molecular docking, biotin pull-down, CETSA, and DARTS, we demonstrate that YAP1 is a direct target of AKBA. YAP1, a core transcriptional co-activator of the Hippo pathway, plays essential roles in cell proliferation, stemness maintenance, and mechanotransduction; its function in immune cells has also drawn increasing attention in recent years [67,68]. Our finding that AKBA binding to YAP1 upregulates USP31 expression, which in turn suppresses NF-κB phosphorylation and CCR5 transcription, extends the functional repertoire of YAP1 from classical proliferation regulation to the control of neutrophil inflammatory polarization, and provides a direct molecular explanation for the immunomodulatory activity of AKBA.

From a therapeutic perspective, this study developed an EGCG-based, NIMP-R14-modified nanodelivery system. EGCG was chosen not only for its ability to self-polymerize into drug-encapsulating coatings and undergo pH-responsive degradation within the weakly acidic fracture microenvironment, but also for its established human safety profile as a natural dietary polyphenol, which offers translational advantages over synthetic polymers. Additionally, EGCG has been reported to possess anti-inflammatory and osteogenic activities that may complement AKBA action, as well as the potential to ameliorate anxiety-like behaviors and reduce systemic stress [69,70]. Modification with the NIMP-R14 aptamer conferred selective neutrophil recognition — confirmed by in vitro uptake assays showing internalization by neutrophils but not by BMSCs, macrophages, or endothelial cells — providing targeting precision that passive delivery strategies lack. In the CRS fracture model, AKBA@E-N exhibited significantly greater efficacy than free AKBA, as reflected by accelerated fracture line blurring, greater callus volume, and more mature bone tissue architecture. This enhanced efficacy likely reflects two synergistic contributions: active neutrophil enrichment increases the local effective concentration of AKBA at the fracture site, while the sustained-release properties of the EGCG shell extend its duration of action.

Several limitations should be acknowledged. First, although we demonstrated that AKBA reduces the TGFβ1^+^CCR5^+^ subset, its effects on other neutrophil subpopulations—particularly those with distinct pro- or anti-inflammatory phenotypes—were not systematically examined. Given the functional heterogeneity of neutrophils in the fracture microenvironment, a broader profiling of neutrophil polarization states would help to fully define the scope of AKBA's immunomodulatory activity. Second, the present findings are derived exclusively from a mouse model; whether the TGFβ1^+^CCR5^+^ neutrophil subset is present in human fracture patients and whether its abundance correlates with anxiety levels or healing outcomes remain to be established in clinical cohorts. Third, the role of the brain-bone axis—specifically, whether AKBA or its nanocarrier formulation exerts direct effects on central stress circuits that could, in turn, modulate peripheral immune responses and bone repair—was not fully explored in this study. Given that AKBA has been reported to possess neuroprotective and potential anxiolytic-like activities, future investigations along this axis may reveal additional layers of therapeutic benefit beyond local immune modulation. Addressing these questions will be critical for translating the current strategy into clinical practice and for fully understanding its mechanism of action in the context of stress-impaired bone healing. In addition, because the formulation was administered locally at the fracture site, the present data support neutrophil-preferential uptake and local retention rather than systemic active targeting. Aptamer surface density, EGCG coating thickness, serum stability, long-term storage stability, and formal lot-to-lot reproducibility were not evaluated experimentally. NIMP-R14 aptamers were linked by physical adsorption and qualitatively confirmed by spectroscopic analysis; all formulations were freshly prepared prior to use. These parameters are important for drug development and scale-up production and should be addressed in future formulation optimization studies before advancing clinical translation.

In summary, this study establishes the TGFβ1^+^CCR5^+^ pathogenic neutrophil subset as a central player in anxiety-associated fracture healing delay, elucidates the molecular mechanism by which AKBA modulates this subset through the YAP1–USP31–NF-κB–CCR5 axis, and validates the feasibility of the NIMP-R14-modified EGCG nanocarrier system for targeted delivery. This strategy offers new theoretical foundations and a candidate approach for clinical intervention in psychological stress-related bone repair disorders.

## 5 Conclusion

This study is the first to show that anxiety delays fracture healing by expanding the TGFβ1^+^CCR5^+^ pathogenic neutrophil subset and reveals that AKBA depletes this subset by targeting the YAP1–USP31–NF-κB–CCR5 axis. Based on this mechanistic framework, we developed an NIMP-R14-modified EGCG nanocarrier system that actively enriches neutrophils and releases AKBA in response to the fracture microenvironment, thereby significantly accelerating bone healing in anxious mice. By combining mechanical dissection with targeted intervention, this strategy offers new therapeutic avenues for psychological stress-related bone repair disorders.

## CRediT authorship contribution statement

**Lizhi Ouyang:** Writing – original draft, Methodology, Investigation, Data curation, Conceptualization. **Yunhua Zhang:** Methodology, Formal analysis, Data curation. **Jiewen Liao:** Formal analysis, Data curation, Conceptualization. **Zhenhe Zhang:** Formal analysis, Data curation. **Xi He:** Methodology. **Ze Lin:** Formal analysis. **Ranyang Tao:** Methodology, Data curation. **Xudong Xie:** Resources. **Fawwaz Al-Smadi:** Investigation. **Wu Zhou:** Funding acquisition. **Hang Xue:** Writing – review & editing, Formal analysis. **Yiqiang Hu:** Writing – review & editing, Methodology. **Dong Yang:** Writing – review & editing, Formal analysis. **Guohui Liu:** Writing – review & editing, Funding acquisition.

## Ethics approval and consent to participate

The animal experiment procedure was approved by the Institutional Animal Care and Use Committee at Tongji Medical College, Huazhong University of Science and Technology (No. 202510443).

## Declaration of competing interest

The authors declare that they have no known competing financial interests or personal relationships that could have appeared to influence the work reported in this paper.
