# Supplementary material for: Aptamer-functionalized EGCG nanocarrier for neutrophil-targeted AKBA delivery promotes anxiety-related fracture healing
Source: Bioact Mater. 2026 Sep 24;69:33–51. doi: 10.1016/j.bioactmat.2026.09.028 (PMC13634590; doi:10.1016/j.bioactmat.2026.09.028)
Supplement: Supplementary file 1 [file mmc1.docx]

**Aptamer-functionalized EGCG nanocarrier for neutrophil-targeted AKBA delivery promotes anxiety-related fracture healing**

**Figure S1. Establishment and validation of a mouse anxiety model induced by chronic restraint stress.** (A) Schematic workflow of anxiety model establishment via 14-day chronic restraint stress CRS and subsequent behavioral test. (B) Summary of overall behavioral test results between the control group and the 14-day CRS-induced anxiety model group. (C) Movement trajectories and statistical analysis of open arm residence time in mice examined by the elevated plus maze EPM. (D) Movement trajectories and statistical analysis of central area residence time in mice assessed by the open field test OFT. (E, F) Plasma concentrations of corticosterone and norepinephrine in Control and CRS mice at multiple time points. (G) Statistical comparison of body weight between the control and anxiety model mice at multiple time points. (H, I, J) Statistical analysis of immobility time in mice tested by the forced swimming test FST; immobility time in mice examined by the tail suspension test TST; and sucrose preference ratio in mice tested by the sucrose preference test SPT at multiple time points. (K, L) Behavioral test of EPM and OFT in CRS mice at multiple time points. n=6 for all mouse experiments.

**Figure S2. Adoptive transfer of TGFβ1+CCR5+ neutrophils impairs fracture healing.** (A) X-ray images at 0, 7, 14, and 21 days after fracture were evaluated to evaluate the macroscopic process of fracture healing in each group. (B) Micro-CT three-dimensional reconstruction images of the fracture callus, along with quantitative analysis of BV/TV and BMD to assess callus formation and bone repair. (C) On day 21 after fracture, Masson's trichrome and H&E staining were performed on fracture callus tissues. n=6 for all mouse experiments.

**Figure S3. 100ns all-atom molecular dynamics simulation analysis of protein-ligand complexes.** (A) Combined RMSD. (B) Radius of gyration Rg. (C) Residue flexibility RMSF. (D) Number of hydrogen bonds. (E) Secondary structure. (F) Solvent accessible surface area. (G) Free energy surface FEL.

**Figure S4. AKBA acts by targeting YAP1 in neutrophils and regulating the NF-κB-CCR5 axis.** (A) The intracellular colocalization of FITC-AKBA and YAP1 in neutrophils in mice was observed by confocal laser microscopy. (B) The specific binding of AKBA to YAP1 was confirmed by drug affinity response target stability assay in neutrophils in mice. (C) Viability of Hoxb8 cells treated with 0, 5, 10, 15 μM AKBA. (D) mRNA levels of NF-κB-CCR5 axis-related molecules in the control, AKBA, and AKBA+si YAP1 groups in Hoxb8 cells. n=3. (E) Protein levels of NF-κB-CCR5 axis-related molecules in the control, AKBA, and AKBA+si YAP1 groups in Hoxb8 cells. (F) Immunofluorescence staining shows that AKBA treatment attenuates NF-κB nuclear translocation in Hoxb8 cells, and siYAP1 reverses this effect.

**Figure S5. AKBA exerts its effects by regulating the YAP1-USP31-NF-κB-CCR5 axis in HL-60.** Four groups were included: HL-60, HL-60+AKBA, HL-60+AKBA+YAP1 overexpression, and HL-60+AKBA+USP31 siRNA. (A) Viability of HL-60 treated with 10 μM AKBA. (B) mRNA levels of *Usp31* and *Ccr5* in HL-60. n=3. (C) Protein levels of USP31, NF-κB, and CCR5 in HL-60. (D) NF-κB nuclear translocation in HL-60 observed by confocal laser microscopy, and analysis of NF-κB nuclear-cytoplasmic fluorescence ratio. (E) Proportions of CCR5⁺ and TGFβ1⁺ HL-60 were analyzed by immunofluorescence staining and analysis of double-positive cell ratio statistics. (F) mRNA levels of osteogenic genes (*Col1a1, Bglap, Runx2*) in BMSCs from co-culture systems. (G) Protein levels of osteogenic markers (COL1A1, OCN, RUNX2) in BMSCs from co-culture systems. (H) Osteogenic activity of BMSCs in co-culture systems assessed by ALP staining. (I) Mineralized nodule formation of BMSCs in co-culture systems was evaluated by Alizarin Red staining.

**Figure S6. Activation status of the TRAF3‑NF‑κB‑CCL5 signaling pathway in BMSC.** (A) mRNA levels of *Traf3* and *Ccl5* in BMSC. (B) Protein levels of TRAF3, NF‑κB, and CCL5 in BMSC. (C) Secretion of CCL5 protein in BMSC culture supernatants was measured by ELISA. (D) Osteogenic activity of BMSCs in co-culture systems assessed by ALP staining. (E) Mineralized nodule formation of BMSCs in co-culture systems was evaluated by Alizarin Red staining. (F) Transwell detects neutrophil migration after different treatments in co-culture system and statistics on the number of migrating cells. (G) Transwell detects Hoxb8 cells migration after different treatments in co-culture system and statistics on the number of migrating cells. n=3.

**Figure S7. Physical characterization and biological functions of AKBA@E-N.** (A–C) XPS, Raman, and FTIR spectra of EGCG, AKBA@E, and AKBA@E‑N, respectively. (D) pH measurement at the fracture site. (E) Mass spectrometry standard curve for AKBA concentration. (F, G) Concentration‑time curve of AKBA in fracture tissues and serum. (H) Immunofluorescence staining for drugs targeting callus sites; scale bars: 200 μm and 40 μm. (I) Flow cytometry gating strategy for LY6G⁺CD11b⁺ double‑positive neutrophils in bone marrow cells. (J) FlowJo analysis of drug enrichment in double-positive neutrophil populations, and average fluorescence intensity statistics. (K) In vivo imaging system (IVIS) images of drug accumulation at 24 h post‑injection, along with quantitative analysis of drug accumulation in the heart, liver, spleen, lung, kidney, and fracture site.

**Figure S8. EGCG has good antibacterial ability.** (A) Schematic diagram of bacterial coating of co-culture system. (B, C) Physical images of MRSA and *E.coli* cultured for 12 h. Quantitative analysis of bacterial count. n=3. (D, E) electron microscopy images of MRSA and *E.coli* cultured for 12 h. Quantitative analysis of the proportion of structurally damaged bacteria. (F) Live/dead staining of MRSA and E.coli .
